# Metabolic syndrome diminishes insulin-induced Akt activation and causes a redistribution of Akt-interacting proteins in cardiomyocytes

**DOI:** 10.1371/journal.pone.0228115

**Published:** 2020-01-29

**Authors:** Huguet V. Landa-Galvan, Emmanuel Rios-Castro, Tatiana Romero-Garcia, Angelica Rueda, Jesus Alberto Olivares-Reyes

**Affiliations:** 1 Departamento de Bioquimica, Cinvestav-IPN, Mexico City, Mexico; 2 Unidad de Genomica, Proteomica y Metabolomica (UGPM), LaNSE-Cinvestav-IPN, Mexico City, Mexico; Université Catholique de Louvain, BELGIUM

## Abstract

Metabolic syndrome (MetS) is a cluster of cardiometabolic risk factors, with insulin resistance as a critical component for its development. Insulin signaling in the heart leads to Akt (also known as PKB) activation, a serine/threonine protein kinase, which regulates cardiac glucose metabolism and growth. Cardiac metabolic inflexibility, characterized by impaired insulin-induced glucose uptake and oxidation, has been reported as an early and consistent change in the heart of different models of MetS and diabetes; however, the evaluation of Akt activation has yielded variable results. Here we report in cardiomyocytes of MetS rats, diminished insulin-induced glucose uptake and Akt activation, evaluated by its impaired mobilization towards the plasma membrane and phosphorylation, and reflected in a re-distribution of its interacting proteins, assessed by label-free mass spectrometry (data are available via ProteomeXchange with identifier PXD013260). We report 45 proteins with diminished abundance in Akt complex of MetS cardiomyocytes, mainly represented by energy metabolism-related proteins, and also, 31 Akt-interacting proteins with increased abundance, which were mainly related to contraction, endoplasmic reticulum stress, and Akt negative regulation. These results emphasize the relevance of Akt in the regulation of energy metabolism in the heart and highlight Akt-interacting proteins that could be involved in the detrimental effects of MetS in the heart.

## Introduction

MetS is a highly prevalent condition characterized by a constellation of physiological and biochemical disorders, such as insulin resistance (IR), obesity and dyslipidemias, which increase the risk of cardiovascular disease [1, 2].

MetS and diabetes mellitus type 2 (DM2) are interrelated conditions that not only often coexist, but also, those people diagnosed with MetS without DM2 are at a significant risk of developing it [1]. Diabetic cardiomyopathy is a specific form of heart disease, characterized in its early stages by diastolic relaxation abnormalities and left ventricular hypertrophy. It is promoted by IR and metabolic inflexibility of heart tissue, compensatory hyperinsulinemia and the progression of hyperglycemia [3–5].

IR condition is a critical factor for MetS and diabetic cardiomyopathy development. Because insulin induces glucose uptake and oxidation, the IR state of the heart affects its capacity to adapt during a period of high energy demand [3, 6]. Insulin exerts its effects by binding to its membrane receptor and inducing its activation by autophosphorylation. Active insulin receptor recruits and phosphorylates the insulin receptor substrate (IRS) proteins, which in turn activates the phosphoinositide 3-kinase (PI3K); this kinase phosphorylates the membrane phospholipid phosphatidylinositol 4,5-bisphosphate (PIP_2_) to form phosphatidylinositol 3,4,5-trisphosphate (PIP_3_), which induces protein kinase B (Akt) mobilization towards the plasma membrane where it is phosphorylated and activated by the phosphoinositide-dependent kinase 1 (PDK1) and mTORC2 [7, 8].

Akt is a serine/threonine kinase that plays a central role in the anti-apoptotic and metabolic actions of insulin, such as glucose uptake, carbohydrate and lipid metabolism, and protein synthesis, among others [9, 10]. In the heart, the three known isoforms of Akt are expressed, with Akt1 and Akt2 being the most abundant [11–13]. Activation of this kinase exerts a cardioprotective effect under diverse pathophysiological conditions by regulating the use of energy substrates [14, 15], inhibiting apoptosis [16, 17], and decreasing endoplasmic reticulum (ER) stress [18].

An impaired insulin-induced glucose uptake has been reported as an early and consistent change in the heart of different models of MetS associated conditions; however, the evaluation of Akt activation has yielded variable results, as discussed by Riehle et al. [19]. It has been suggested that in pre-diabetic states, an Akt hyperactivation stage may occur before the insulin´s capacity to activate this kinase declines; therefore, variability in Akt activation results is probably due to the stage or severity of metabolic alterations in the studied model [20].

Although a diminished Akt activation in the heart has been associated with metabolic inflexibility and diabetic cardiomyopathy development [3, 19], sustained long-term Akt activity also has deleterious effects; for instance, it induces cardiac hypertrophy, which may lead to heart failure [21], so its activity must be finely regulated.

Akt activity can be regulated by phosphorylation and also by interacting proteins [22]. Akt-interacting proteins have been classified into three groups: 1) substrates that are directly phosphorylated by Akt, 2) Akt activity regulators, and 3) proteins regulated simply by binding to Akt [23, 24]. More than a hundred Akt-interacting proteins have been described, which emphasizes the important role of this kinase in a wide variety of cellular processes [9].

Here, we aimed to evaluate insulin-induced glucose uptake and Akt activation in cardiomyocytes of MetS rats, and also quantify changes in the abundance of proteins that form a complex with Akt by using a co-immunoprecipitation (CoIP) and label-free mass spectrometry approach, in order to provide proteomic support to the Akt activation status and to explore the molecular mechanisms related to Akt regulation that could be involved in the heart IR condition and the diabetic cardiomyopathy development.

We found an impaired glucose uptake and diminished Akt activation in insulin-stimulated cardiomyocytes of MetS rats. These conditions were reflected in the mass spectrometry results, which revealed a diminished Akt interaction with energy metabolism-related proteins, and also an increased Akt interaction with proteins related to contraction and ER stress response in cardiomyocytes of MetS rats.

## Materials and methods

### Ethics statement

In the present study, we employed Wistar rats as an experimental model of metabolic syndrome; these animals were euthanized with an intraperitoneal injection of sodium pentobarbital (100 mg/kg) and heparin (1000 U/kg). All procedures were carried out under the ethical guidelines of the Mexican Official Norm (NOM-062-ZOO-1999) and the National Institutes of Health Guide for the Care and Use of Laboratory Animals (NIH publication updated in 2011). The Institutional Bioethical Committee approved it for Care and Handling of Laboratory Animals at the Cinvestav-IPN (approved CICUAL protocol No. 0105–14) [25].

### Experimental strategy

A general workflow scheme is presented in S1 Fig (Supporting information).

### Development of MetS in rats

Weaning male Wistar rats (approx. 22 days old) with body weights of 74.0 ± 2.0 g were randomly divided into two groups: control and MetS. Control group rats received tap water for drinking, and MetS rats received water with 30% sucrose for four months. Both groups were fed with balanced PicoLab Rodent Diet 20 (LabDiet) *ad libitum* and kept under controlled conditions of temperature (19–23 °C) and light-dark cycle (12h/12h) until euthanasia. The night before sacrifice (8 hours fasting), food pellets were removed from all rat cages, and 30% sucrose solution was replaced by water in the case of MetS rats. On the day of euthanasia, fasting glucose was measured in a blood sample from the tail vein prior to anesthetic injection, while triglycerides and HDLc levels were determined in a blood sample from the thoracic cavity, with the CardioCheck PA analyzer (PTS Diagnostics). A blood sample was taken from the thoracic cavity (after anesthetic injection (100 mg/kg sodium pentobarbital) and heart excision) to determine serum insulin concentration; serum was obtained and stored at −70 °C until assayed using an ultrasensitive rat insulin ELISA (#80-INSRTU-E01, ALPCO). Besides, body weight, heart weight, epididymal fat weight, and tibia length were recorded.

### Glucose tolerance test

The glucose tolerance test was performed as previously reported [26]. Briefly, after 8-hour fast, rats were injected intraperitoneally with glucose (2 g/kg), local lidocaine (5%) was applied on the tail, and glucose levels in tail vein blood were measured at 0, 15, 30, and 60 min after injection.

### Cardiomyocytes isolation

Isolation of ventricular myocytes was conducted as previously reported, with some modifications [27]. Briefly, rats were given an intraperitoneal injection of sodium pentobarbital (100 mg/kg) and heparin (1000 U/kg). The thoracic cavity was opened, and the heart excised and rapidly placed in ice-cold Tyrode solution containing (in mM): 130 NaCl; 5.4 KCl; 0.4 NaH_2_PO_4_; 0.5 MgCl_2_; 22 glucose, and 25 HEPES (pH 7.4 with NaOH). The aorta was cannulated above the aortic valve, and the heart was retro-perfused with Tyrode solution supplemented with 0.23 mM EGTA at 37 °C for 2 min. Digestion solution containing 300 U/ml collagenase type II (Worthington Biochemical Corp.) in Tyrode solution supplemented with 0.1 mM CaCl_2_ was then perfused until the aortic valve was digested (increased outflow). The heart was taken down from the cannula, the ventricles were dissected and placed in a 15-ml plastic tube containing digestion solution supplemented with BSA (1 mg/ml), and gently triturated for 3 min with a plastic pipette to disperse myocytes. The resulting cell suspension was subjected to centrifugation for 3 min at 170 x *g*. The cell pellet was suspended in Tyrode solution supplemented with increasing CaCl_2_ concentrations (in mM: 0.25, 0.5, and 0.8) and centrifuged after suspension in each solution at the same speed (170 x *g*). Finally, the cell pellet was suspended in a storage solution containing Tyrode solution supplemented with 1 mM CaCl_2_.

### [^3^H]-2-deoxy-glucose uptake assay

Insulin-induced glucose uptake in cardiomyocytes was determined by measuring [^3^H]-2-deoxy-glucose ([^3^H]-2-DG) uptake, as previously described, with some changes [28]. Cardiomyocytes were washed twice with 1mM CaCl_2_ Tyrode solution without glucose (used in all the washing steps) and were left to stabilize for 20 min at 37°C. Cells were then incubated with 100 nM insulin for 30 min; glucose uptake was quantified by exposing the cells to 1 μCi/ml [^3^H]-2-DG + 0.1 mM 2-DG in Tyrode solution without glucose for 15 min at 37 °C. At the end of the 15 min period, the supernatant was aspirated rapidly, and the cells were washed three times. Cardiomyocytes were lysed in 0.1 N NaOH with SDS 0.1%, and the associated radioactivity was determined by liquid scintillation counting (Beckman LS6500) and normalized according to the total protein content. Nonspecific uptake was determined in the presence of 20μM cytochalasin B. Each experiment was assayed by duplicate. Results were expressed in fmol of ^3^H-2-dG/mg of protein/min.

### Immunostaining

Cardiomyocytes were cultured in glass coverslips pretreated with laminin (#L2020, Sigma-Aldrich, 1:70 in PBS). After one hour, cells were stimulated with 100 nM insulin for 2 min, washed with cold PBS, and fixed with 4% paraformaldehyde solution for 10 minutes. Cells were washed with PBS (3 x 5 min) and incubated with blocking solution (0.2% Triton in PBS + 5% normal goat serum) for 30 min. Cells were then incubated with Akt antibody (sc-8312, Santa Cruz, 1:200 dilution) overnight at 4° C; all the antibodies were diluted in PBS + 0.2% Triton + 1% normal goat serum. The next day, cells were washed with 0.2% Triton in PBS (3 x 5 min) and incubated with caveolin-3 antibody (C38329, Transduction Laboratories, 1:200) for 3 hours at room temperature. Cells were then washed and incubated with secondary antibodies, Alexa Fluor488-conjugated goat anti-rabbit IgG (A31627, Invitrogen, 1:200) and Rhodamine Red-X-conjugated goat anti-mouse IgG (R6393, Invitrogen, 1:200) for 2 hours each, at room temperature in the dark. Finally, cells were washed with PBS (3 x 5 min), and the coverslips were mounted on slides using ProLong Gold antifade reagent (P36934, Invitrogen).

Cells were imaged using a Zeiss LSM700 confocal microscope (Carl Zeiss Microscopy, Oberkochen, Germany). Colocalization of Akt and caveolin-3 was quantified with the weighted colocalization coefficient calculated by the Zeiss ZEN 2010 software (Carl Zeiss Microscopy), which uses the same equation as the Manders coefficient [29]; however, the value for each pixel ranges from 0 to 1 depending on its intensity.

### Insulin treatments

Isolated cardiomyocytes were cultured in 6-well plates, placed in an incubator and left to adhere for 2 hours. Then, cells were stimulated for 10 min with 0.1, 1, 10, 100 and 1000 nM insulin. After stimulation, cells were washed with ice-cold storage solution (Tyrode + 1 mM CaCl_2_) and lysed with 100 μl of 1× Laemmli sample buffer (0.05 M Tris Base, 2% SDS, 10% glycerol, 0.1% bromophenol blue, 5% β-mercaptoethanol). Protein was quantified using RC-DC Protein Assay Kit (Bio-Rad). These samples were stored at −20 °C until analyzed by Western blot.

### CoIP assay

Isolated cardiomyocytes were stimulated with 100 nM insulin for 10 min and lysed with cold non-denaturing lysis buffer (in mM: (50) Tris HCl pH 7.4, (150) NaCl, (2) orthovanadate, (1) NaF, (2) EDTA; 1% NP-40, 10% glycerol, containing protease inhibitors). The lysate was centrifuged for 15 min at 4 °C, and the supernatant was transferred to a new tube. For pre-clearing, 10 μl of protein A-Sepharose (Zymed) were added, the tube was placed in rotary motion at 4° C for 1 hour, centrifuged for 2 min at 13,000 rpm and the supernatant was transferred to a new tube. For CoIP, 2.5 μl of polyclonal Akt 1/2/3 antibody (sc-8312, Santa Cruz) plus 20 μl of protein A-Sepharose were added, the tube was placed in rotary motion overnight and centrifuged for 2 min at 13,000 rpm. The Sepharose-bound immune complexes were washed three times with 500 μl of cold lysis buffer to remove non-specifically bounded proteins. Finally, immune complexes were suspended in Laemmli buffer with 10% β-mercaptoethanol, and protein concentration was determined using the RC-DC Protein Assay Kit (Bio-Rad).

### Western blot

Samples were sonicated at 40 kHz for 20 s, heated at 99 °C for 5 min and centrifuged at 14,000 rpm for 5 min at room temperature; 20 μg of protein (from insulin response assays samples) or 10 μg of protein (from Akt CoIP samples for mass spectrometry validation), were electrophoresed on SDS-PAGE (10–12% gels) and separated proteins were electro-transferred to PVDF membranes. Blots were blocked with 5% Blotto solution (sc-2325, Santa Cruz) in TBST for 1 hour, and incubated overnight at 4 °C with primary antibodies. Blots were washed 3 times and incubated with horseradish peroxidase-conjugated secondary antibodies for 1 h at room temperature. Antibody binding was visualized with ECL (Enhanced Chemiluminescence) reagent (Immobilon^™^ Millipore Corporation) and quantified with ImageJ software (National Institutes of Health). Primary antibodies: Akt 1/2/3 (sc-8312, 1:5000), pAkt Ser473 (sc-7985, 1:3000), 14-3-3 ζ (sc-1019, 1:5000) and Hsp60 (sc-13115, 1:1000), were purchased from Santa Cruz Biotechnology; pAkt Thr308 (9275S, 1:3000) from Cell Signaling; GAPDH (AM4300, 1:35000), from Thermo Fisher Scientific. Actin (mouse monoclonal, 1:8000) was provided by Dr. J.M. Hernandez from Cell Biology Department, CINVESTAV, Mexico. Secondary antibodies: HRP-goat anti-mouse IgG (115-035-003, 1:10000) and HRP-goat anti-rabbit IgG (111-035-144, 1:10000) were purchased from Jackson ImmunoResearch.

### Statistical analysis

GraphPad PRISM^™^ version 6.0 software was used for statistical analysis and plotting of the glucose tolerance test, glucose uptake, Akt-caveolin-3 colocalization and Western blot results. ANOVA was used for comparisons of more than two groups, and t-test for unpaired data in the case of two-group comparisons. Values of *P* < 0.05 were considered statistically significant.

### Label-free mass spectrometry

#### Sample preparation and mass spectrometry analysis

Protein samples (30 μg each) from CoIP assays of insulin-stimulated cardiomyocytes (N = 3 control rats, N = 3 MetS rats), were loaded on a 10% SDS-PAGE. In-gel digestion of samples was done according to Shevchenko et al. [30] using 20 ng/μl trypsin (mass spectrometry grade from Sigma-Aldrich). Resulting peptides were desalted using Pierce C-18 spin columns (Thermo Scientific) and then injected in a mass spectrometer Synapt G2-S*i* (Waters, Milford, MA) in MS^E^ mode in order to calculate the AUC of the total ion chromatogram (TIC) and thus normalize the injection in the nanoUPLC. The same amount of tryptic peptides in each condition were loaded into Symmetry C18 Trap V/M precolumn (180 μm X 20 mm, 100 A° pore size, 5 μm particle size) and desalted using 0.1% formic acid (FA) in H_2_O as mobile phase A, and 0.1% FA in ACN as mobile phase B, under the following isocratic gradient: 99.9% mobile phase A and 0.1% of mobile phase B at a flow of 5 μl.min^-1^ during 3 min. Afterward, peptides were loaded and separated on HSS T3 C18 Column (75 μm X 150 mm, 100 A° pore size, 1.8 μm particle size), using an UPLC ACQUITY M-Class with the same mobile phases under the following gradient: 0 min 7% B, 121.49 min 40% B, 123.15 to 126.46 min 85% B, 129 to 130 min 7% B, at a flow of 400 nl.min^-1^ and 45 °C. The spectra data were acquired in a mass spectrometer with ESI and ion mobility separation (IMS) Synapt G2-S*i*, using data-independent acquisition (DIA) approach through HDMS^E^ mode (Waters). Tune page for the ionization source was set with the following parameters: 2.75 kV in the sampler capilar, 30 V in the sampling cone, 30 V in the source offset, 70 °C for source temperature, 0.5 Bar for nanoflow gas and 150 L hr^-1^ for purge gas flow. Two chromatograms were acquired (low and high energy chromatograms) in positive mode in a range of m/z 50–2000 with a scan time of 500 ms. No collision energy was applied to obtain the low energy chromatogram while for the high energy chromatograms, the precursor ions were fragmented in the transfer using a collision energy ramp of 19–55 V. Generated *.raw files were analyzed using DriftScope v2.8 software (Waters) to selectively apply quasi-specific collision energies based on the drift time for each peptide detected in the mass spectrometer. A *.rul file was generated and used to apply specific collision energy for every peptide detected in the UDMS^E^ mode (instead of a linear ramp as in the HDMS^E^ mode). With the same chromatographic and source conditions used in the HDMS^E^ mode, tryptic peptides for each condition were injected three times (technical replicates), and the UDMS^E^ mode was applied accordingly.

#### Data analysis

The MS and MS/MS intensities contained in the generated *.raw files were normalized, aligned, compared and relatively quantified using Progenesis QI for Proteomics software v3.0.3 (Waters) against a *Rattus norvegicus* *.fasta database (downloaded from UniProt, 29971 protein sequences, last modified on December 1st, 2017), concatenated with its reversed database. The parameters used for protein identification were: trypsin as cut enzyme and one missed cleavage allowed; carbamidomethyl (C) as a fixed modification and oxidation (M), amidation (C-terminal), deamidation (Q, N) or phosphorylation (S, T, Y) as variable modifications; peptide and fragment tolerance were set to automatic, minimum fragment ion matches per peptide: 2, minimum fragment ion matches per protein: 5, minimum peptide matches per protein: 1, and false discovery rate ≤ 4%. Synapt G2-S*i* was calibrated with [Glu1]-fibrinopeptide, [M+2H]^2+^ = 785.84261 at ≤ 1 ppm. The results generated from Progenesis Software were exported to *.xlsx files to verify two levels of data quality control for label-free experiments (peptide and protein level) according to the figures of merit (FOM) described by Souza et al. [31]. All proteins considered with differential interaction with Akt presented at least a ratio ±1.2 (expressed as a base 2 logarithm); it means that these proteins had at least ± 2.29-absolute fold change interaction (diminished or increased) with Akt in cardiomyocytes of MetS *vs* control rats. The ratio was calculated dividing the average MS signal response of the three most intense tryptic peptides (Hi3) of each well-characterized protein in the MetS experiments by the Hi3 of each protein in the control assays.

#### Bioinformatic analysis and protein classification

Functional enrichment analysis was performed independently for proteins with increased or diminished interaction with Akt using STRING database (version 11.0) [32], which is based on straightforward over-representation analysis using hypergeometric tests [33]. Proteins were classified according to the biological process related to their function using STRING classification based on GO database as a guide; the classification was further refined considering the specific tissue context, and the results were plotted using PRISM^™^, version 6 (GraphPad Software).

#### Data accessibility

The mass spectrometry proteomics data have been deposited to the ProteomeXchange Consortium [34] (http://proteomecentral.proteomexchange.org) via the PRIDE [35] partner repository with the dataset identifier PXD013260.

## Results

A scheme depicting the number of animals distributed over the performed analysis is shown in S2 Fig (Supporting Information).

### MetS rats

MetS rats developed obesity evidenced by a significant increase in body weight (~1.4-fold increase *vs* control rats) and epididymal fat (~2.4-fold increase), without changes in growth assessed by tibia length; they also developed hyperinsulinemia and dyslipidemias (hypertriglyceridemia and decreased HDL cholesterol) without significant changes in fasting blood glucose (Table 1).

**Table 1:** Corporal and seric parameters of control and MetS rats.

Group	Body weight (g)	Heart weight (g)	Epididymal fat (g)	Tibia (cm)	Triglycerides (mg/dL)	HDL (mg/dL)	Glucose (mg/dL)	Insulin (ng/mL)
**Control**	415 ± 5	1.8 ± 0.04	7.3 ± 0.4	4.9 ± 0.05	60±3	44±2	64 ± 4	4.9 ± 0.6
**MetS**	592 ± 12***	2.1 ± 0.06**	17.5 ± 1***	5 ±0.03	137 ± 10***	31 ± 2**	75 ± 6	17.1 ± 2.3***

Results are presented as mean ± SEM.

**P < 0.01,

***P < 0.001.

N = 10–13 for both groups.

**Fig 1:**
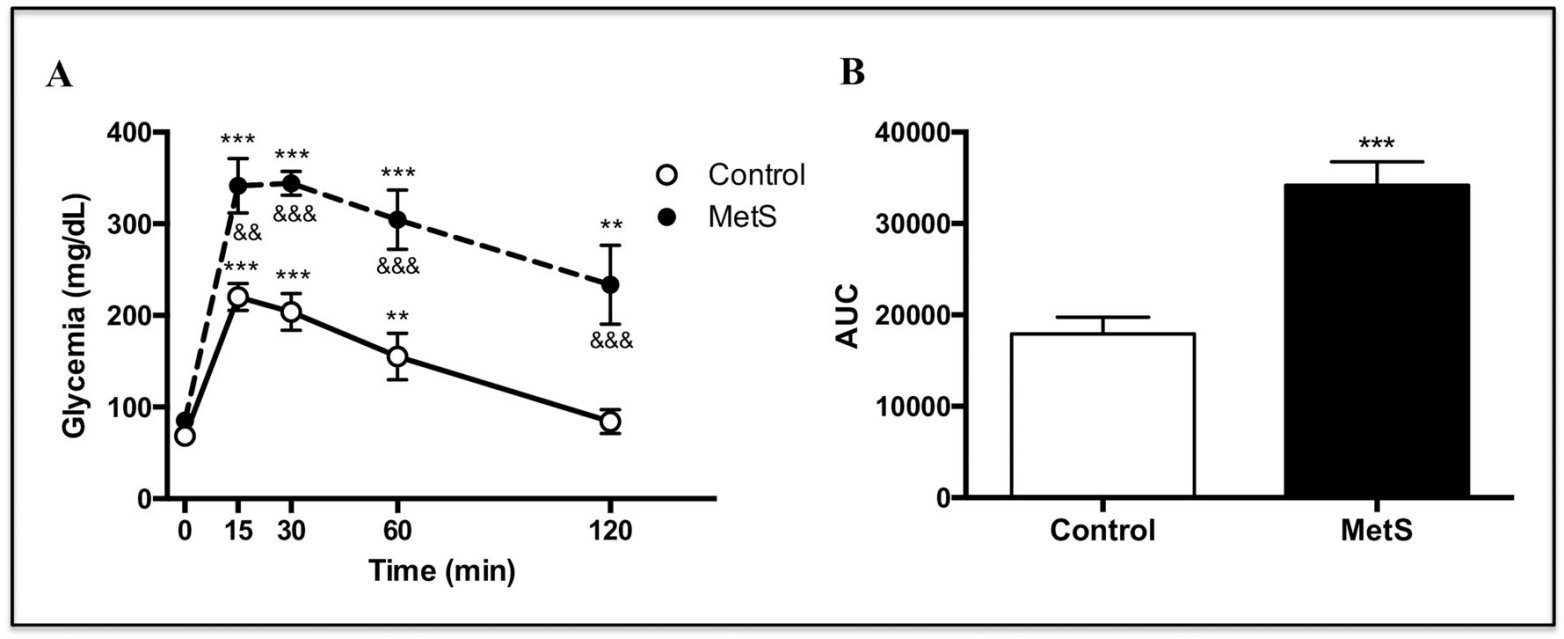
Glucose tolerance test. A) Glucose determinations performed in control and MetS rats at 15, 30, 60 and 120 minutes after intraperitoneal injection of glucose (2g / kg). N = 6, for both groups. **P < 0.01, ***P < 0.001 *vs* basal (0 min). ^&&^*P* <0.01, ^&&&^*P* < 0.001 *vs* control rats. B) Area under the curve of the glucose tolerance test. ***P < 0.001 *vs* control rats.

In the glucose tolerance test, MetS rats presented higher glycemic peaks and were unable to return to basal blood glucose levels after two hours of the glucose bolus injection. (Fig 1A). AUC as an estimate of glycemia during the complete test was also significantly increased in MetS rats, which is indicative of a systemic insulin-resistant state (Fig 1B).

### Impaired insulin-induced glucose uptake in MetS cardiomyocytes

**Fig 2:**
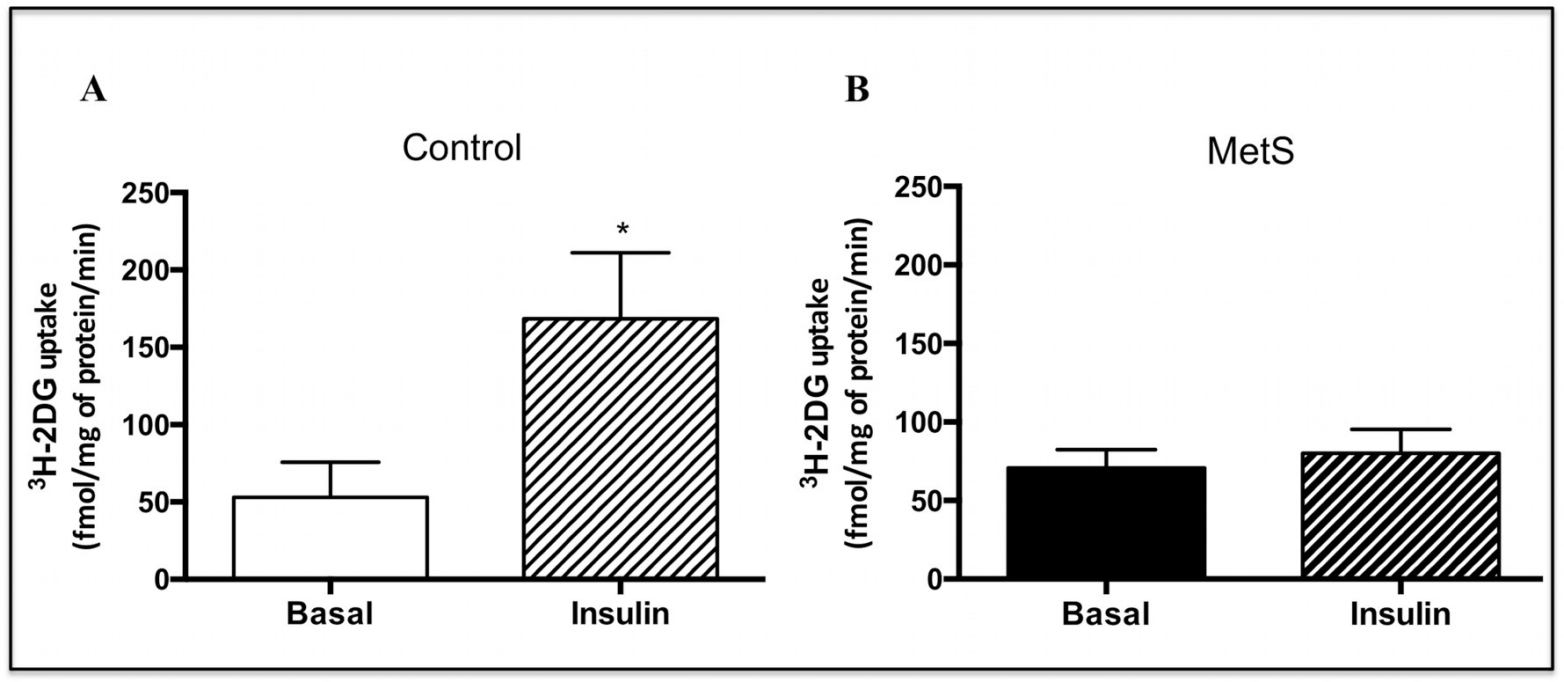
^3^H-2-deoxy-D-glucose uptake. Cardiomyocytes of A) Control and B) MetS rats were stimulated with 100 nM insulin for 30 min, and the ^3^H-2-deoxy-D-glucose uptake was measured in a scintillation counter. N = 5, for both groups. *P < 0.05.

We performed glucose uptake assays in isolated cardiomyocytes to evaluate insulin response, and we found a significant 3.2-fold increase in control cardiomyocytes (mean ± SEM in fmol/mg of protein/min: Basal: 53 ± 23; Insulin: 168 ± 43; Fig 2A). Conversely, in MetS cardiomyocytes glucose uptake was not significantly increased in response to insulin (mean ± SEM in fmol/mg of protein/min: Basal: 70 ± 12; Insulin: 80 ± 15; Fig 2B), indicating that the systemic IR state of the MetS rats, was also reflected in a diminished insulin response in isolated cardiomyocytes.

### Impaired insulin-induced Akt activation in MetS cardiomyocytes

**Fig 3:**
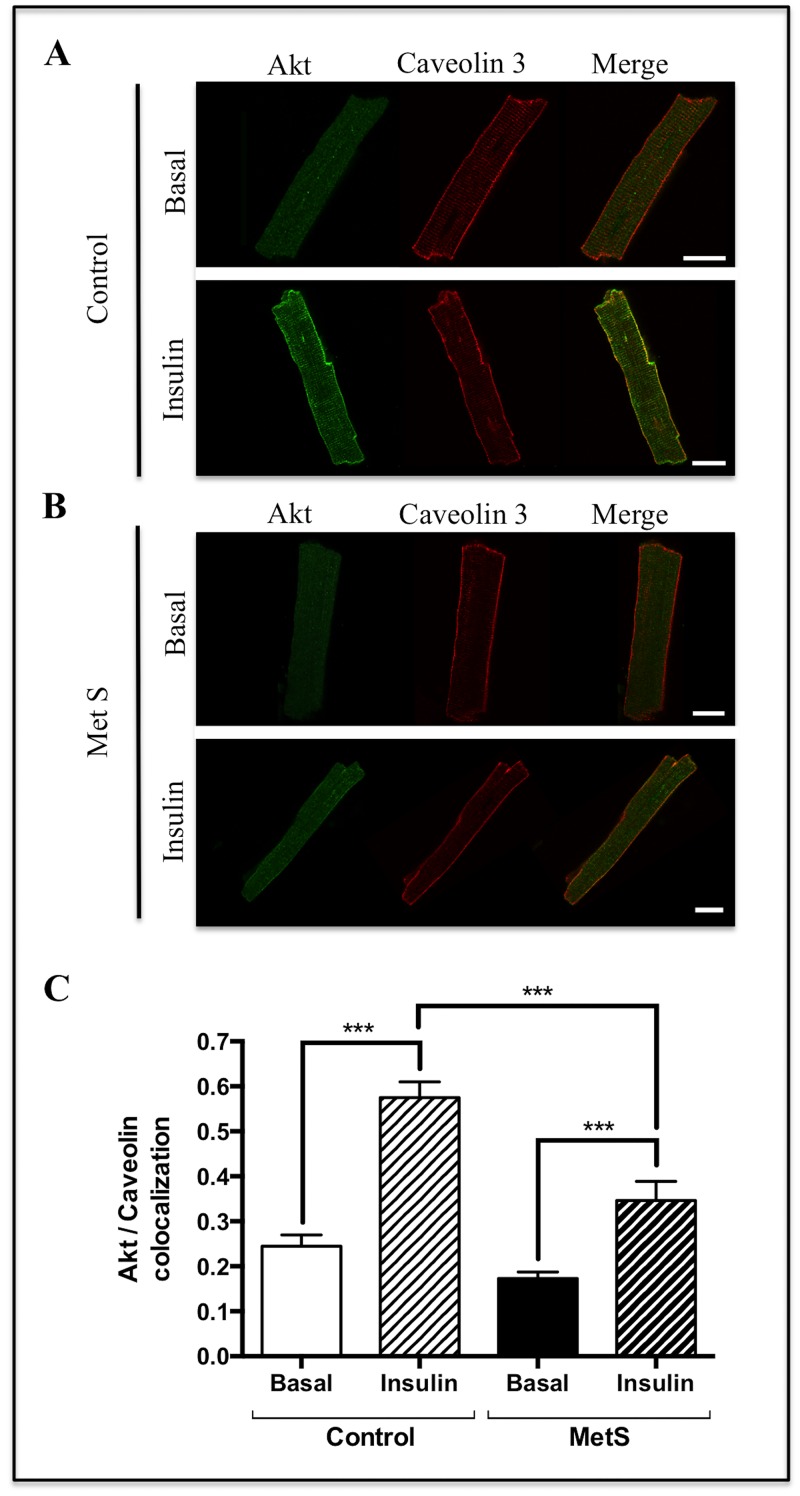
Akt mobilization towards caveolin-3 regions in response to insulin. A) Control and B) MetS cardiomyocytes were stimulated with 100 nM insulin for 2 min, and the Akt (green) and caveolin-3 (red) localization was evaluated by immunostaining using confocal microscopy. C) Akt/caveolin-3 weighted colocalization coefficient. Scale bar = 20 μm. N = 3 rats, n = 30 cardiomyocytes, for both groups. ***P < 0.001.

After confirming the insulin resistance state in cardiomyocytes, evidenced by impaired glucose uptake, as reported in different models [36, 37], we aimed to evaluate the insulin-induced Akt activation in cardiomyocytes isolated from our MetS model. As a first step for Akt activation, this kinase translocates towards the plasma membrane. Ebner et al. reported in HeLa and MCF-7 cells that insulin induces a rapid and transient accumulation of a fraction (5%–15%) of cytosolic Akt at the plasma membrane, with the maximal accumulation occurring at times less than 5 min [38]. We evaluated Akt mobilization towards the plasma membrane by assessing its colocalization with caveolin-3, a muscle-specific integral membrane protein [39], after insulin stimulation for 2 min in control (Fig 3A) and MetS cardiomyocytes (Fig 3B) and found a significantly diminished colocalization coefficient in MetS (0.34 ± 0.04) *vs* control (0.57 ± 0.03) cardiomyocytes (*P* < 0.001, Fig 3C), without significant changes in the basal colocalization without insulin. Plasmalemmal caveolin-3 fluorescence intensity was not modified in MetS condition (S3 Fig, Supporting Information).

**Fig 4:**
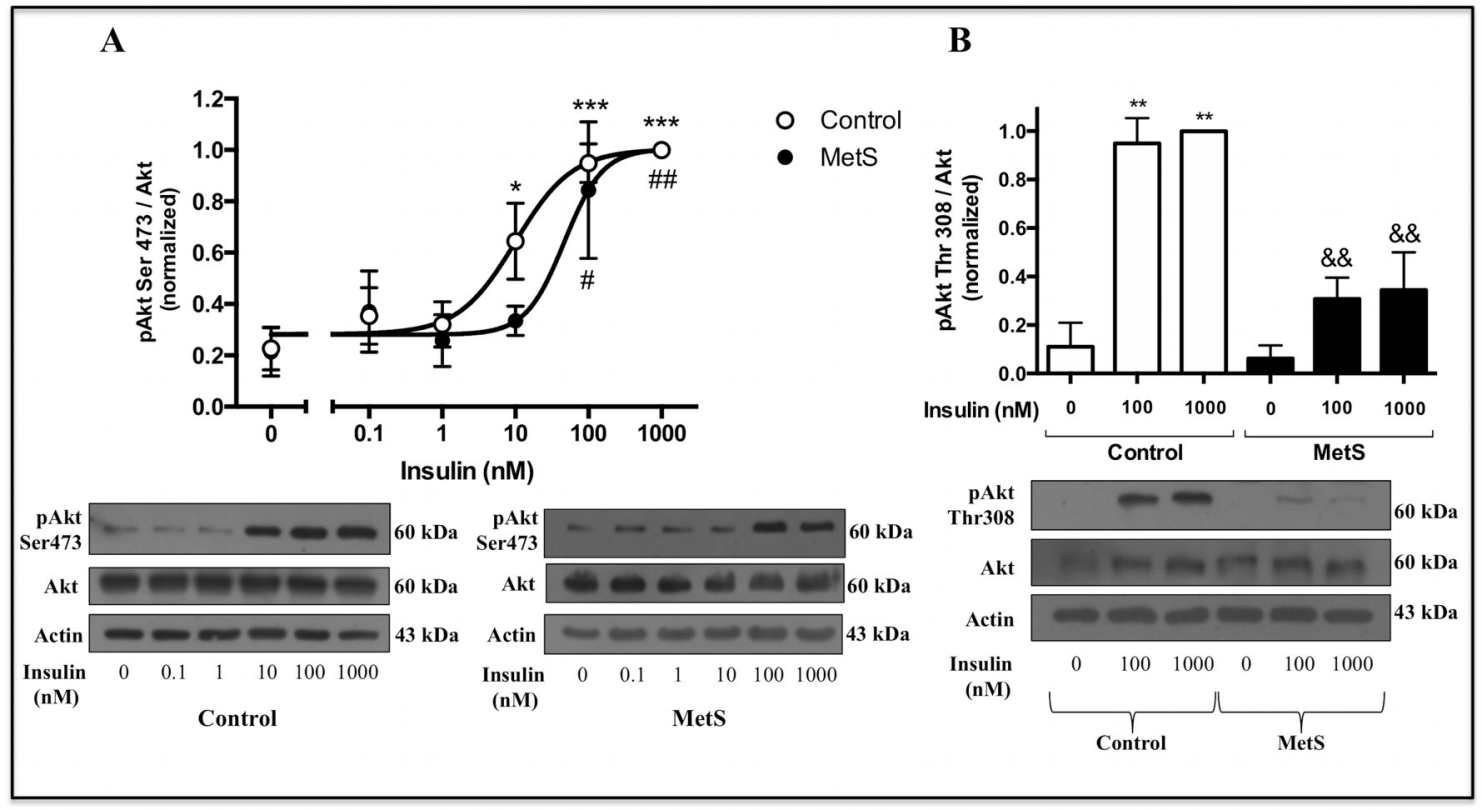
Akt phosphorylation in response to insulin. Control and MetS cardiomyocytes were stimulated with the indicated insulin concentrations for 10 min, and Akt phosphorylation in its main activation residues was evaluated by Western blot. A) pAkt Ser473. Data were normalized with the maximum value being 1 for both curves (N = 5 control, 3 MetS. *P < 0.05, ***P < 0.001 *vs* basal in control cardiomyocytes. ^#^*P* < 0.05, ^##^*P* < 0.01 *vs* basal in MetS cardiomyocytes); B) pAkt Thr308 (N = 3, for both groups. **P < 0.01 *vs* basal. ^&&^*P* < 0.01 *vs* control rat cardiomyocytes).

After mobilization towards the plasma membrane, Akt is activated by phosphorylation in threonine 308 and serine 473 residues, by PDK1 and mTORC2 kinases, respectively [8]. We stimulated cardiomyocytes with increasing insulin concentrations (0.1–1000 nM) and found a significant increase in Akt phosphorylation at serine 473 at higher concentration of insulin in MetS cardiomyocytes (100 nM insulin) than in control cardiomyocytes (10 nM insulin), showing a right shift in the concentration-response curve (control EC_50_ = 10 nM, MetS EC_50_ = 47 nM) which indicates a diminished insulin sensitivity in terms of Akt activation in MetS cardiomyocytes (Fig 4A). We also evaluated Akt phosphorylation at threonine 308 after stimulation with two maximal insulin concentrations, 100 and 1000 nM, and found a significant reduction of 67% and 66%, respectively, in MetS cardiomyocytes (Fig 4B).

### Redistribution of Akt-interacting proteins in MetS cardiomyocytes

To characterize Akt-interacting proteins, we performed CoIP using Akt antibody and identified associated proteins using label-free mass spectrometry.

#### Peptide quality control

According to our peptide quality control analysis and after removing ions with charge state z = 1^+^ (Panel A in S4 Fig, Supporting Information), we were able to detect 20,016 peptides in the entire study: 91.30% (18,274) of them had an error of ± 10 ppm (Panel B in S4 Fig, Supporting Information). These peptides were classified by peptide-matched type (Panel C in S4 Fig, Supporting Information): 39.1% of them were denominated PepFrag1 and 33.1% as VarMod (peptides containing some variable modification). PepFrag1 and VarMod peptides were distributed throughout the *m/z* full range with a mass error no greater than ±10 ppm (Panel D in S4 Fig, Supporting Information). These peptides were the most reliable in our study since those were identified during database search pass-1 and pass-2 by the algorithm of Progenesis software. This algorithm considers fourteen physicochemical parameters that contribute to the peptide score, such as complete enzymatic digestion, a high number of ions products matched, and a good correlation of the sum of product ions intensity with the precursor ions intensity, among others. PepFrag2 peptides (16.3% of total peptides) were identified during database search pass-3, without restrictions on the ion intensity [40]. Besides, 10.4% of peptides had one missed cleavage, only 0.8% were fragmented at source, and finally, 0.3% presented neutral loss of H_2_O or NH_3_. These results showed a high-quality at peptide level, justifying their use to identify and quantify proteins. Moreover, these results proved an adequate calibration of the mass spectrometer and efficient enzymatic digestion.

#### Protein quality control and quantitative analysis

To profile the proteomic of Akt-interacting proteins from control and MetS cardiomyocytes, label-free quantitative MS-based proteomic analysis was performed.

A total of 360 proteins were detected in this study, with an average of 13.21 peptides per protein; of them, 43 proteins were only identified but not quantified, 8 proteins were exclusively found in a specific condition (4 proteins in control condition, and 4 proteins in MetS condition), and finally, 309 proteins were quantified with an average of 12.93 peptides per protein. All these results are available in S1 File (Supporting Information).

The false discovery rate (FDR) of the study, calculated based on target/decoy database method [41], was 0.1% at the peptide level and 0.4% at the protein level, considering 5 peptides per protein.

**Fig 5:**
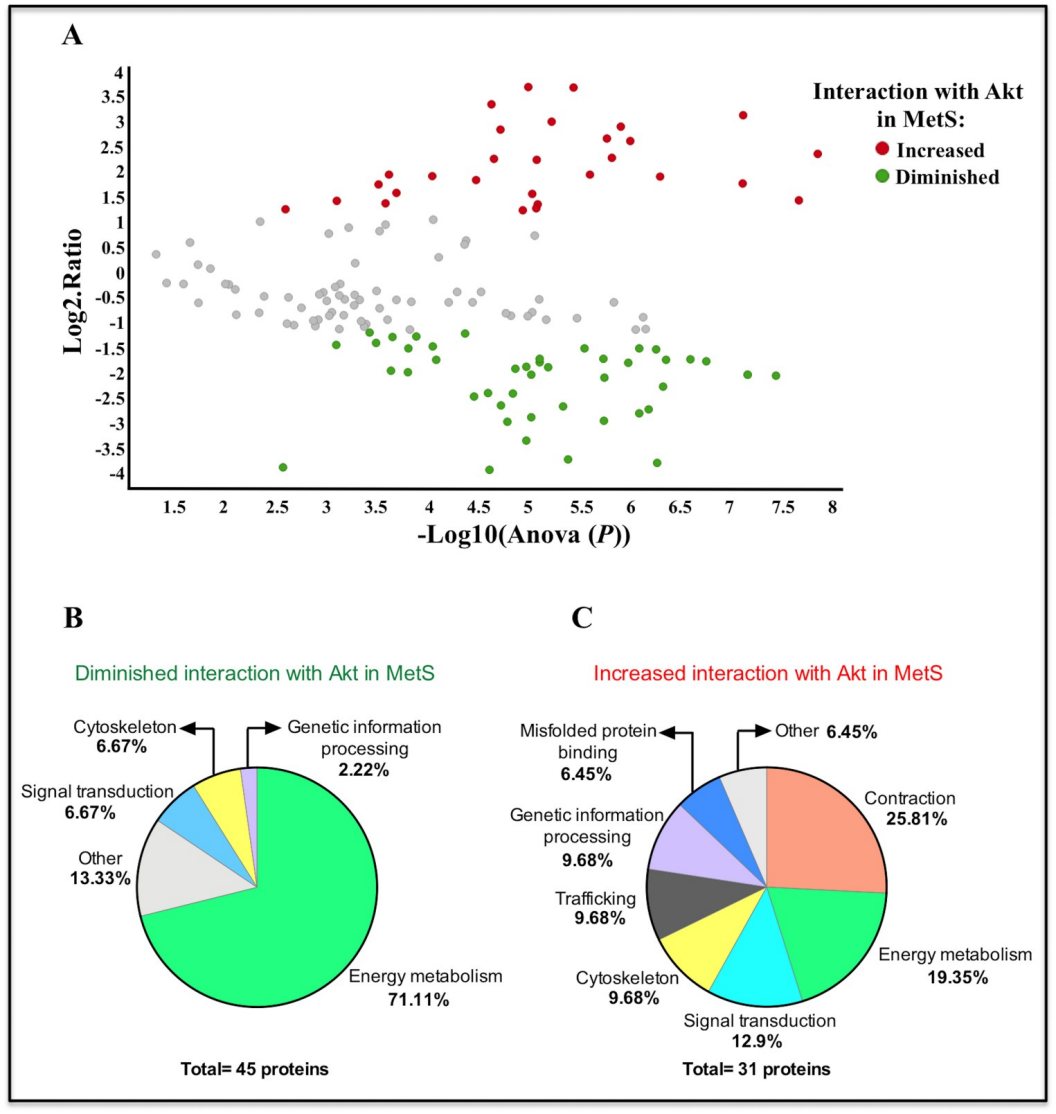
Proteins differentially interacting with Akt in MetS cardiomyocytes. Control and MetS cardiomyocytes were stimulated with 100 nM insulin for 10 min; Akt CoIP was performed, and the relative abundance of proteins interacting with Akt was assessed by label-free mass spectrometry. A) Volcano plot representing all filtered proteins: gray circles correspond to the proteins that are in complex with Akt but did not change their abundance between the MetS and control samples (69), red circles correspond to the proteins with increased abundance in Akt complex in MetS (29), green circles correspond to the proteins with diminished abundance in Akt complex in MetS (44). The X-axis corresponds to the *P*-value of each detected protein in the technical triplicate (values represented as -Log10), and Y-axis corresponds to the ratio of the average Hi3 intensities (MetS/C) of the technical triplicate for each detected protein (values are represented as Log2). Proteins were classified according to the biological process related to their function using STRING based on the GO database, and pie charts depicting this functional classification were constructed for B) proteins with diminished interaction with Akt in MetS and C) proteins with increased interaction with Akt in MetS.

Quantified proteins had a dynamic range of ~5.5 orders of magnitude (base 10 logarithm) (S5 Fig, Supporting Information); this is an abundance difference of around three hundred thousand times between the less and more abundant proteins, which shows a high sensitivity of the MS. Quantified proteins were filtered based on the following parameters and conditions: coefficient of variation ≤ 0.20; at least 2 peptides per protein; at least 1 unique; ANOVA ≤ 0.05; only proteins detected and quantified in the three independent experiments for both conditions were considered, and reversed proteins were excluded. Finally, 142 quantified proteins were filtered and scattered in a volcano plot (Fig 5A).

There was no abundance difference in 69 Akt-interacting proteins between control and MetS cardiomyocytes (gray circles in Fig 5A; protein list in S1 Table, Supporting Information). In the volcano plot, 44 proteins were detected with diminished abundance in Akt complex of MetS cells (green circles in Fig 5A), and also, 1 of the 4 proteins exclusively found in control (non-detectable levels in MetS) met the criteria of having at least 2 detected peptides, which adds for a final total of 45 proteins with diminished interaction with Akt in MetS cardiomyocytes (protein list in Table 2). The functional enrichment assay indicated that the most represented biological process among these proteins was the carboxylic acid metabolic process considering the GO database classification (GO:0019752, FDR: 7.88e-17), and in the pie chart depicting the functional classification (Fig 5B), it can be observed that the proteins that decrease their interaction with Akt in MetS condition are mainly those related to energy metabolism (71.1%).

**Table 2:** Akt-interacting proteins with diminished abundance in MetS.

Accession	Description	Peptide count	Unique peptides	Confidence score	Max fold change
P63102	14-3-3 protein zeta/delta	2	2	9.0	Exclusive in Control
D3ZSD8	Transmembrane protein 143	2	1	11.2	-15.3
B1WBL5	Cilia and flagella-associated protein 52	2	2	9.7	-14.8
Q68FU3	Electron transfer flavoprotein subunit beta	2	2	13.0	-14.0
O55171	Acyl-coenzyme A thioesterase 2, mitochondrial	3	2	18.4	-13.3
P41562	Isocitrate dehydrogenase [NADP] cytoplasmic	2	1	18.7	-10.2
Q6P6R2	Dihydrolipoyl dehydrogenase, mitochondrial	7	6	40.3	-7.9
P97700	Mitochondrial 2-oxoglutarate/malate carrier protein	6	5	49.6	-7.8
P16036	Phosphate carrier protein, mitochondrial	8	8	49.0	-7.4
Q5BJZ3	Nicotinamide nucleotide transhydrogenase	36	35	168.2	-7.0
P15650	Long-chain specific acyl-CoA dehydrogenase	11	10	88.7	-6.6
Q5XIT9	Methylcrotonoyl-CoA carboxylase beta chain	3	2	20.1	-6.4
P03889	NADH-ubiquinone oxidoreductase chain 1	4	4	27.0	-6.3
E9PT79	Translin	2	1	10.3	-5.6
D3ZB81	Solute carrier family 25 member 31	14	1	48.6	-5.3
Q09073	ADP/ATP translocase 2	22	2	78.1	-5.3
P26284	Pyruvate dehydrogenase E1 component subunit alpha	10	6	46.4	-4.8
P00507	Aspartate aminotransferase, mitochondrial	14	14	73.6	-4.3
P49432	Pyruvate dehydrogenase E1 component subunit beta	12	12	81.0	-4.2
P04797	Glyceraldehyde-3-phosphate dehydrogenase	7	1	47.6	-4.1
Q05962	ADP/ATP translocase 1	38	17	129.4	-4.1
Q60587	Trifunctional enzyme subunit beta, mitochondrial	10	9	54.5	-4.0
P18886	Carnitine O-palmitoyltransferase 2, mitochondrial	7	7	39.9	-3.9
P09605	Creatine kinase S-type, mitochondrial	11	1	65.7	-3.8
P56574	Isocitrate dehydrogenase [NADP], mitochondrial	36	32	202.8	-3.7
P42123	L-lactate dehydrogenase B chain	16	15	79.9	-3.7
Q8VHF5	Citrate synthase, mitochondrial	13	13	57.3	-3.5
D4A7L4	NADH dehydrogenase (Ubiquinone) 1 beta subcomplex, 11 (Predicted)	7	7	47.8	-3.5
P15999	ATP synthase subunit alpha, mitochondrial	46	42	238.8	-3.4
Q9ER34	Aconitate hydratase, mitochondrial	34	27	179.9	-3.3
D4ACD3	Ubiquitin specific protease 25 (Predicted)	2	1	9.4	-3.3
D4A565	NADH dehydrogenase (Ubiquinone) 1 beta subcomplex, 5 (Predicted), isoform CRA_b	9	9	57.1	-3.3
Q64428	Trifunctional enzyme subunit alpha, mitochondrial	21	18	120.3	-3.3
P04636	Malate dehydrogenase, mitochondrial	21	18	118.9	-3.3
D3ZTG6	SFI1 centrin-binding protein	9	6	50.6	-2.9
Q9QXQ0	Alpha-actinin-4	7	5	35.5	-2.9
P45953	Very long-chain specific acyl-CoA dehydrogenase	6	5	35.2	-2.9
F1LZW6	Solute carrier family 25 member 13	16	9	86.9	-2.9
D3ZHC7	RCG43605, isoform CRA_a	2	2	8.7	-2.8
Q9WVC1	Slit homolog 2 protein (Fragment)	3	3	14.5	-2.7
Q4G069	Regulator of microtubule dynamics protein 1	2	2	9.3	-2.7
P51868	Calsequestrin-2	11	11	74.1	-2.4
B1WCA0	Ppm1d protein	2	2	8.2	-2.4
P05065	Fructose-bisphosphate aldolase A	8	7	35.9	-2.3
P09760	Tyrosine-protein kinase Fer	2	2	5.1	-2.3

In contrast, a final total of 31 proteins were detected with increased abundance in Akt complex of MetS cardiomyocytes (red circles in Fig 5A, protein list in Table 3), considering 2 proteins exclusively found in MetS cells that met the selection criteria. The most represented biological process among these proteins was the muscle contraction (GO: 0006936, FDR: 2.05e-07), as can be observed in Fig 5C, in which contraction related proteins accounted for 25.81% of all the proteins with increased abundance in Akt complex of MetS cardiomyocytes.

**Table 3:** Akt-interacting proteins with increased abundance in MetS.

Accession	Description	Peptide count	Unique peptides	Confidence score	Max fold change
Q9ESN0	Protein Niban	2	1	15.3	Exclusive in MetS
A0A0G2JVL8	Isoleucyl-tRNA synthetase	2	1	9.1	Exclusive in MetS
M0RD07	Uncharacterized protein	2	1	10.3	12.8
P63259	Actin, cytoplasmic 2	76	10	309.2	12.7
P04692	Tropomyosin alpha-1 chain	34	1	152.2	10.1
D4ADF6	Zinc finger FYVE-type-containing 16	3	3	28.1	8.7
D3ZCV0	Actinin alpha 2	11	4	60.6	8.0
D3ZAN6	Poly(A) polymerase gamma	2	2	4.9	7.4
P02564	Myosin-7	364	2	1968.3	7.1
P06686	Sodium/potassium-transporting ATPase subunit alpha-2	3	1	20.8	6.3
P06761	78 kDa glucose-regulated protein	5	4	26.8	6.1
P68035	Actin, alpha cardiac muscle 1	115	44	479.7	5.1
A0A0G2K6A9	Protein RUFY3	5	4	27.9	4.8
P50753	Isoform 2 of Troponin T, cardiac muscle	2	2	14.4	4.8
P19527	Neurofilament light polypeptide	2	1	15.8	4.7
D3ZAF6	ATP synthase subunit f, mitochondrial	2	2	10.3	3.8
Q7TP48	Adipocyte plasma membrane-associated protein	3	3	14.5	3.8
Q6P6V0	Glucose-6-phosphate isomerase	3	3	14.6	3.8
G3V885	Myosin-6	426	1	2228.8	3.7
A0A0G2JXE0	Histone H2B	7	5	39.3	3.5
P16409	Myosin light chain 3	52	47	249.4	3.4
P46462	Transitional endoplasmic reticulum ATPase	4	3	28.9	3.3
Q63065	Pyruvate dehydrogenase (acetyl-transferring) kinase isozyme 1, mitochondrial	3	3	15.6	3.0
D3ZAA9	MAGUK p55 subfamily member 2	2	1	10.8	2.9
D3ZD09	Cytochrome c oxidase subunit	5	4	35.0	2.7
P10860	Glutamate dehydrogenase 1, mitochondrial	2	2	19.0	2.7
P63039	60 kDa heat shock protein, mitochondrial	4	4	24.0	2.6
Q5XIH7	Prohibitin-2	19	18	108.0	2.5
B1H267	Sorting nexin-5	2	2	8.6	2.4
Q3T1K5	F-actin-capping protein subunit alpha-2	3	3	16.1	2.4
F1LM47	Succinate-CoA ligase [ADP-forming] subunit beta, mitochondrial	3	3	16.8	2.3

Abundance of proteins differentially interacting with Akt in MetS cardiomyocytes *vs* control (normalized fold change) is represented in a heat map (S6 Fig, Supporting Information). Proteins are classified by their main physiological function.

**Fig 6:**
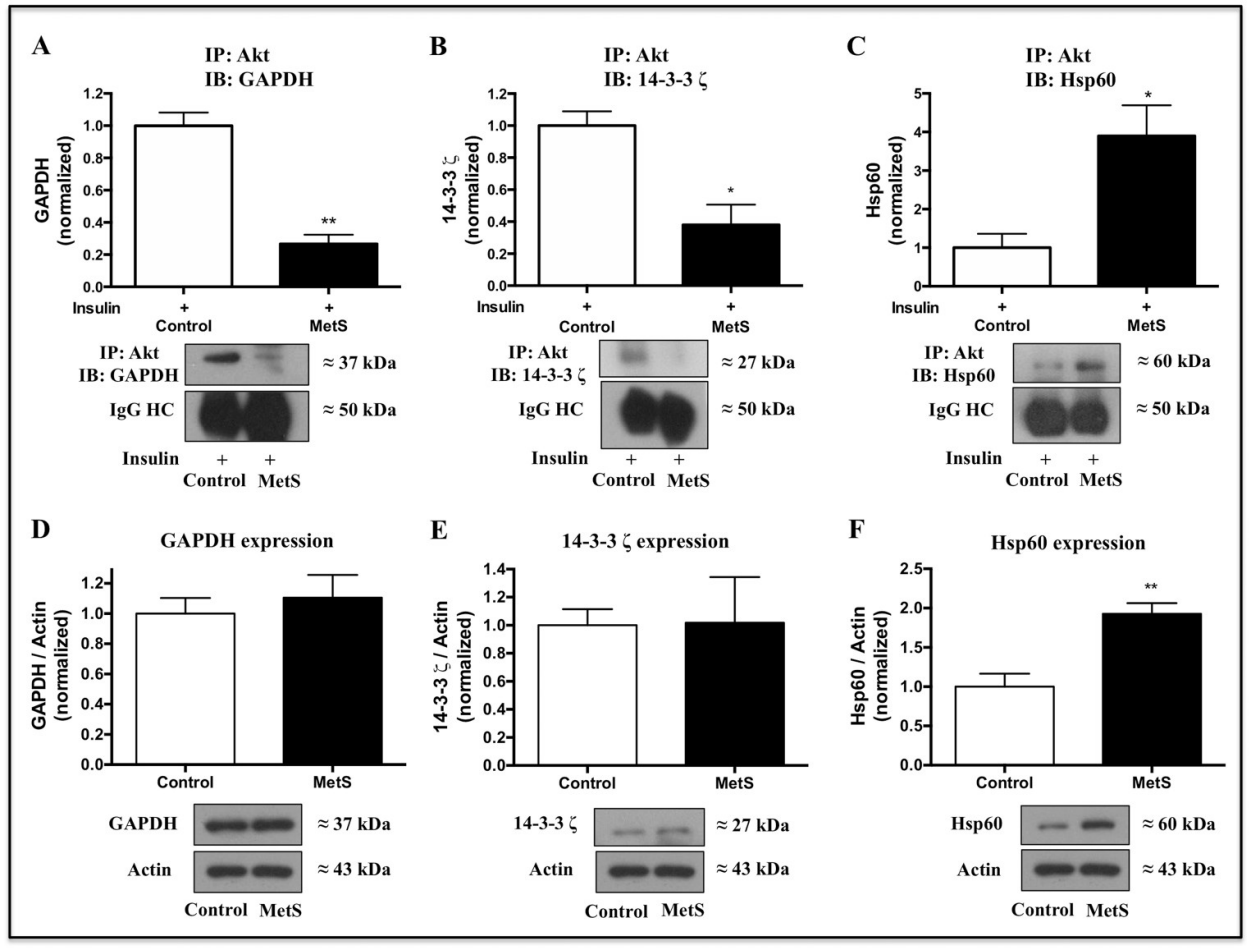
Validation of a subset of Akt-interacting proteins. Akt interaction with A) GAPDH, B) 14-3-3 ζ, and C) Hsp60 was evaluated in insulin-stimulated control and MetS cardiomyocytes (100 nM insulin for 10 min) by Akt immunoprecipitation and Western Blot. Representative image of three independent experiments. IgG heavy chain (HC) is presented as loading control. *P < 0.05, **P < 0.01 *vs* control. Expression levels of D) GAPDH, E) 14-3-3 ζ, and F) Hsp60 were evaluated in cardiomyocytes lysates by Western Blot. Representative image of four independent experiments. **P < 0.01 *vs* control.

As confirmatory data, we evaluated Akt interaction with two proteins found with diminished abundance, glyceraldehyde-3-phosphate dehydrogenase (GAPDH) and 14-3-3 ζ, and one with increased abundance, Hsp60, by Akt immunoprecipitation and Western Blot in independent samples. In agreement with the mass spectrometry results, we found a diminished Akt interaction with GAPDH (Fig 6A) and 14-3-3ζ (Fig 6B), as well as increased interaction with Hsp60 (Fig 6C).

In order to assess whether changes in total levels of these Akt-interacting proteins could explain the modifications observed in Akt complexes, we evaluated their expression by Western Blot in total cardiomyocytes lysates. In the case of GAPDH (Fig 6D) and 14-3-3 ζ (Fig 6E), there was no change between control and MetS expression levels; on the other hand, Hsp60 was significantly increased in MetS cardiomyocytes (Fig 6F).

## Discussion

### MetS rats

In sucrose-fed MetS models, different factors such as rat strains, as well as the amount, duration, and mode of sucrose feeding (pellet or drinking water), influence the development of different metabolic disorders [42]. About the sucrose delivery mode, Singh et al. [43] reported that male Sprague Dawley rats fed with high sucrose diet (55% of energy as sucrose) supplied by pellets for 15 weeks, did not develop obesity, hypertriglyceridemia or insulin resistance, unlike reported in other studies [44], however, by supplying sucrose in the drinking water (32% sucrose), rats developed obesity and insulin resistance in 8 to 10 weeks [43]. Those results are in agreement with our model of sucrose supplementation in drinking water, in which the rats became obese with an increased epididymal fat and body weight (Table 1) [25].

The weight gain in our model [25] contrasts with other studies using the same animal model in which no weight differences [45–47] or much lower weight increases were found [48]. Regardless of the variability in the characteristics developed, sucrose-induced MetS has been a frequently studied model to elucidate molecular alterations of this condition [45–49], and the sucrose-drinking protocol used in our study induced central characteristics of MetS, such as obesity, hyperinsulinemia, hypertriglyceridemia, and insulin resistance (Table 1 and Fig 1).

### Impaired insulin-induced glucose uptake and Akt activation in MetS cardiomyocytes

Isolated cardiomyocytes from MetS rats showed an impaired insulin response, evidenced by the absence of a significant increase in glucose uptake in response to this hormone (Fig 2). Impaired insulin-induced glucose uptake in heart has been reported in different studies, mainly conducted in Zucker *fa/fa* obese rats [37, 50], a model widely used for the study of myocardial IR [51], and also in sucrose-fed rats [36]. However, in those studies, a diminished basal glucose uptake (without insulin stimulation) was reported, contrasting with our results, where no changes in this parameter were found. Mazumder et al. [52] reported unchanged basal rates of glucose uptake and a completely blunted insulin-induced glucose uptake in cardiomyocytes isolated from ob/ob mice, results similar to ours. Impaired insulin-induced glucose uptake is considered as an early alteration in myocardial IR that may occur even before the activation of Akt in response to insulin is affected [20]. We found a diminished Akt activation in response to insulin assessed by its mobilization towards the plasma membrane (Fig 3) and its phosphorylation status in the two main activation residues, serine 473 and threonine 308, in MetS cardiomyocytes (Fig 4). In agreement with our results, some studies conducted in similar models have also shown an impaired insulin-induced serine 473 phosphorylation of Akt in the heart; however, in contrast to our findings, they found an increased basal phosphorylation state [53, 54].

### Redistribution of Akt-interacting proteins in MetS cardiomyocytes

Providing proteomic support to the diminished Akt activation in MetS cardiomyocytes, we found a diminished interaction of Akt with proteins related to energy metabolism and some positive regulators, and also an increased interaction with proteins associated with contraction, ER stress, and IR (Fig 5, Tables 2 and 3). Akt interaction changes with functional relevant proteins are further discussed below.

#### Akt-interacting proteins with diminished abundance in MetS

The PI3K/Akt pathway has a critical role in glucose metabolism, and the dysregulation of glucose uptake and oxidation in the heart of sucrose-fed rats has already been described [55]. The diminished interaction of Akt with energy metabolism-related proteins found in our study further emphasizes the relevance of this process in MetS and the critical role of Akt in heart metabolism.

In this context, it has been shown that insulin induces Akt-GAPDH interaction, which enhances GAPDH glycolytic activity in H9c2 cardiomyocytes [56]. Nevertheless, we found a diminished abundance of GAPDH in complex with Akt in MetS cardiomyocytes (Fig 6A), suggesting that Akt-induced glycolysis up-regulation is impaired. We did not find changes in GAPDH expression levels (Fig 6D); however, this protein interacts with phosphorylated/active Akt [56, 57], thus, diminished Akt activation in MetS condition could be involved in the decreased Akt-GAPDH interaction.

Pyruvate dehydrogenase (PDH) is another energy metabolism-related protein that is regulated by insulin. It has been reported that insulin induces Akt interaction with pyruvate dehydrogenase (PDH) complex in mitochondria, and this interaction up-regulates PDH enzymatic activity in cardiomyocytes [58]. Interestingly, Carvajal et al. [55], using a similar experimental model to ours, reported a diminished level of cardiac PDH in its active form. In agreement with those results, we found a diminished abundance of PDH in complex with Akt in MetS cardiomyocytes. In this context, it has been reported that the signaling activity of the PI3K/Akt pathway decreases PDH inactivation by PDH kinases [59]. Hence, it is interesting to have found PDH Kinase 1, an isoform highly expressed in heart, with an increased abundance in the Akt complexes of MetS cardiomyocytes. Although it is worthy of mentioning that the overexpression of the isoform PDH Kinase 4 has been associated with IR and diabetic cardiomyopathy development [60, 61], our data unveil the participation of additional PDH kinase isoforms in the establishment of the metabolic disturbances in the MetS heart.

Mitochondria play a critical role in the regulation of myocardial metabolism and function, and it has been shown that Akt activation in mitochondria increases ATP production and minimizes oxygen consumption [58]. Furthermore, mice fed with a high fat/high sucrose diet have a diminished cardiac insulin-induced Akt and ATP synthase activation in mitochondria [62]. Therefore, in the context of MetS, our work brings to light new Akt-interacting proteins related to energy metabolism and with diminished abundance to further study in the future.

In a different context, it has been reported that the capture of phosphoproteins by 14-3-3 proteins regulates insulin actions; and the study of the interaction dynamics of these proteins allows improving the understanding of how insulin can induce diverse cellular responses [63]. Interestingly, we found a diminished interaction of 14-3-3 ζ isoform with Akt in MetS hearts (Fig 6B); this isoform has been reported as an Akt-interacting protein [64] and as a substrate of Akt [65], and it has been shown that its overexpression induces Akt activation in epithelial cells [66, 67]. Under this evidence, it is plausible that the positive regulation that 14-3-3 ζ exerts on Akt is impaired in MetS cardiomyocytes.

Because no difference in 14-3-3 ζ expression levels was found in MetS *vs* control cardiomyocytes (Fig 6E), other factors like Akt diminished activation, localization, or post-translational modifications, could be involved in the diminished Akt-14-3-3 ζ interaction.

We also found that protein phosphatase magnesium-dependent 1 delta (PPM1D), which participates in the anti-apoptotic effect of Akt, decreases in MetS cardiomyocytes. Particularly, it has been reported that Akt stabilizes PPM1D since a diminished Akt expression reduces PPM1D half-life significantly; likewise, when overexpressing Akt, the amount of PPM1D increases [68]. Considering we found a decreased abundance of PPM1D in Akt complex, this result supports the diminished Akt activation found in MetS cardiomyocytes.

#### Akt-interacting proteins with increased abundance in MetS

Akt-actin interaction is involved in the regulation of Akt intracellular distribution and the dynamic reorganization of actin cytoskeleton [69, 70]; however, a direct Akt regulation of the contractile machinery has not been described. Because Akt is mobilized to the plasma membrane, mitochondria, and nucleus after insulin stimulation, it is conceivable that in the MetS condition, a diminished Akt activation in response to insulin increases the presence of this kinase in the cytoplasm. This different Akt subcellular location could be a determinant factor for promoting an increased interaction with proteins related to contraction. It is fair to mention that CoIP experiments reveal direct and indirect interactions, which means that the proteins described in this work are forming a complex with Akt but not necessarily have direct interaction with this kinase.

The protein Niban is a known substrate of Akt that is involved in its anti-apoptotic effects by inducing p53 degradation [71]. We found Niban with increased abundance in the Akt complexes of MetS cardiomyocytes. Conceivably, the decreased Akt interaction with proteins related to energy metabolism led to an enrichment of proteins related to other Akt functions in response to insulin, as the anti-apoptotic effect.

Isoleucyl tRNA synthetase has a non-classical role as an intracellular amino acid sensor that directly interacts with mTORC1 and induces its activation, leading to protein synthesis [72]. This protein was also found with increased abundance in complex with Akt in MetS cells, and it is likely to be involved in the anabolic effect of insulin; however, there is no evidence of direct interaction with Akt.

The ER stress results from an imbalance between protein load and folding capacity that leads to the unfolded protein response, and it has been associated with IR development [73, 74]. It has been reported that inducing ER stress increases expression levels of 78 kDa glucose-regulated protein, also known as Bip or GRP78, and induces its interaction with non-phosphorylated (inactive) Akt in the plasma membrane [75]. Interestingly, we found an increased interaction of this protein with Akt in MetS, suggesting that the cardiomyocytes are in the process of ER stress and that Akt interacting with this protein is probably in an inactive state.

PHB2 is a transcriptional repressor inhibited by interaction with Akt [23]. It has been reported that it also plays a role in regulating insulin response at Akt level: knockdown of PHB significantly increases Akt phosphorylation, while the activation of Akt induces a down-regulation in PHB content, and a diminished Akt function increases PHB content [76, 77]. We found an increased PHB2-Akt interaction in MetS, which suggests a possible increased PHB2 expression as a result of the diminished Akt activation in the MetS condition.

Hsp60 has been linked to IR as a circulating factor; human adipocytes release it, and its circulating levels are increased in plasma of obese subjects. Hsp60 decreases insulin-induced Akt phosphorylation in serine 473, assessed in human subcutaneous adipocytes and skeletal muscle cells [78].

In the heart context, it has been reported that in sucrose-fed rats, the expression of Hsp60 in myocardium increases when animals develop IR, thiazolidinediones treatment reduces Hsp60 levels, and when the rats develop diabetes with insulin deficiency, the expression of Hsp60 is drastically reduced. Chen and collaborators suggest that this over-expression in response to IR occurs as a protection mechanism against cardiac damage [79].

Incubating cardiomyocytes with insulin induces a dose-dependent increase in Hsp60 levels; this protein increases IGF-1 receptor expression and activity, and Hsp60 downregulation has been proposed to be a relevant mechanism for diabetic cardiomyopathy development [80, 81]. We found higher expression levels of Hsp60 in MetS cardiomyocytes (Fig 6F), which could explain the observed increased abundance of Hsp60 in Akt complexes (Fig 6C); Hsp60 up-regulation suggests that MetS myocardium is activating compensatory and protective mechanisms as part of the insulin-resistant state.

Akt isoforms have been related to different physiological functions in the heart. While Akt1 is proposed to be more involved in the regulation of cardiac growth [12], Akt2 has been shown to play a significant role in regulating cardiac metabolism and survival [82, 83]. In our experiments, we used an anti-pan-Akt antibody (Akt 1/2/3); therefore, we cannot rule out the possibility that the observed changes in the MetS condition are associated with diminished expression or activity of a specific Akt isoform.

Because Akt2 activation is particularly related to insulin-induced glucose uptake [82], a lack of insulin response in terms of glucose uptake in cardiomyocytes of MetS rats suggests an Akt2 diminished expression or activity.

In agreement with our data, it has been reported that Akt2 activity selectively decreases in the heart of streptozotocin-induced diabetic mice [84]. Also, chronic Akt2 impaired signaling could be involved in the deterioration of cardiac function, since double knockout (KO) of Akt2 and AMPK induces premature aging-related cardiac structural and contractile changes [85].

On the other hand, the regulation of Akt inactivation is also important since Akt2 activity has also been associated with cardiac function impairment in metabolic disorders. In this respect, it has been reported that in mice under high-fat diet (HFD), Akt2 cardiac expression increases, and Akt2 KO protects against HFD-induced contractile dysfunction by a mechanism that involves rescuing defective autophagosome maturation [86]. Furthermore, Akt2 KO has also been shown to prevent cardiac mitochondrial injury induced by paraquat, an oxidative stress triggering herbicide [87].

Considering the above-discussed data, impairment of Akt signaling in MetS cardiomyocytes might be an adaptive mechanism in response to metabolic or oxidative disorders induced by sucrose consumption; however, further experiments are needed to confirm this hypothesis.

Finally, it is important to mention that all experiments were consistently performed in isolated cardiomyocytes, in order to explore Akt activation and protein-Akt interactions exclusively in this cell type, under controlled conditions. However, energy turnover and demand in cardiomyocytes within a working heart, as well as availability of different energy substrates, could have a relevant effect on Akt signaling in vivo, which we need to consider in future research.

## Conclusions

In summary, we found that in insulin-stimulated cardiomyocytes of MetS rats, an impaired glucose uptake and Akt activation occurs, and also Akt decreases its interaction with proteins related to energy metabolism and some positive regulators, and increases its interaction with proteins related to contraction, ER stress, and IR, a redistribution manner that reflects the impaired glucose metabolism and Akt signaling in the heart. Further studies are needed to determine whether the observed changes are due only to the reported lower Akt activation, or if other factors are involved, such as changes in the expression levels of the different Akt isoforms, or those of its interacting proteins.

## Supporting information

S1 Fig General workflow scheme. Male Wistar rats received tap water (control group) or 30% sucrose as drinking water (MetS group) for 4 months. Cardiomyocytes were isolated and insulin response was assessed by [3H]-2-Deoxy-D-glucose uptake assays. To evaluate insulin-induced Akt activation, the localization of this kinase was determined by immunostaining and its phosphorylation in the two main activation residues, serine 473 and threonine 308, by Western Blot. Identification and relative abundance of proteins interacting with Akt were evaluated by label-free mass spectrometry, in order to provide proteomic support to its activation status and the insulin resistance state of the cardiomyocytes.
(TIF)

S2 Fig Workflow of animals in the study. Scheme depicting the number of control and MetS rats distributed over the performed analyses.
(TIF)

S3 Fig Plasmalemmal caveolin-3 immunofluorescence intensity. Bar chart representing average caveolin-3 immunofluorescence intensity in 10 regions of interest (ROIs) with an area of 10 μm^2^, selected in the plasmalemmal region of 10 cardiomyocytes (10 ROIs per cell) from each experimental condition. AU = arbitrary units.
(TIF)

S4 Fig Peptide quality control. **A)** Movement of the ions inside of the mobility cell. Ions with charge state *z* = 1^+^ (*blue dots*) were discarded in this study, only ions with charge state of *z* = 2^+^ or higher (*peptides indicated by green*, *red and yellow dots*) were used to identified and quantify proteins. **B)** Histogram representing 20,016 peptides which 91.3% had an error of ±10 ppm, no peptide used for the identification and quantification of proteins exceeded ± 38 ppm. **C)** Peptide match type classification. PepFrag 1 peptides and VarMod peptides represent 72.2% of the total ions. **D)** PepFrag 1 and VarMod peptides (*black dots*) are distributed through the full m/z range, in no more than ± 10 ppm. These peptides are the most reliable for protein identification.
(TIF)

S5 Fig Dynamic range of quantified proteins. In white are represented the proteins in the control sample and in black the proteins in MetS. X-axis corresponds to the number of identified and quantified proteins (ID’s); Y-axis corresponds to the average of the Hi3 intensities in the technical triplicate for each detected protein (values are represented as Log10).
(TIF)

S6 Fig Heat map of proteins differentially interacting with Akt. Heat map representation of proteins with abundance changes in Akt immunocomplexes in MetS cardiomyocytes *vs* control. Proteins are classified by main physiological function.
(TIF)

S1 File Excel data file. (XLSX)

S1 Table Proteins with unchanged interaction with Akt in MetS cardiomyocytes. (DOCX)

S1 Raw Images Original images for blots. (PDF)
