# Supplementary figures and images for: Metabolic syndrome diminishes insulin-induced Akt activation and causes a redistribution of Akt-interacting proteins in cardiomyocytes
Source: PLoS One. 2020 Jan 29;15(1):e0228115. doi: 10.1371/journal.pone.0228115 (PMC6988918; doi:10.1371/journal.pone.0228115)

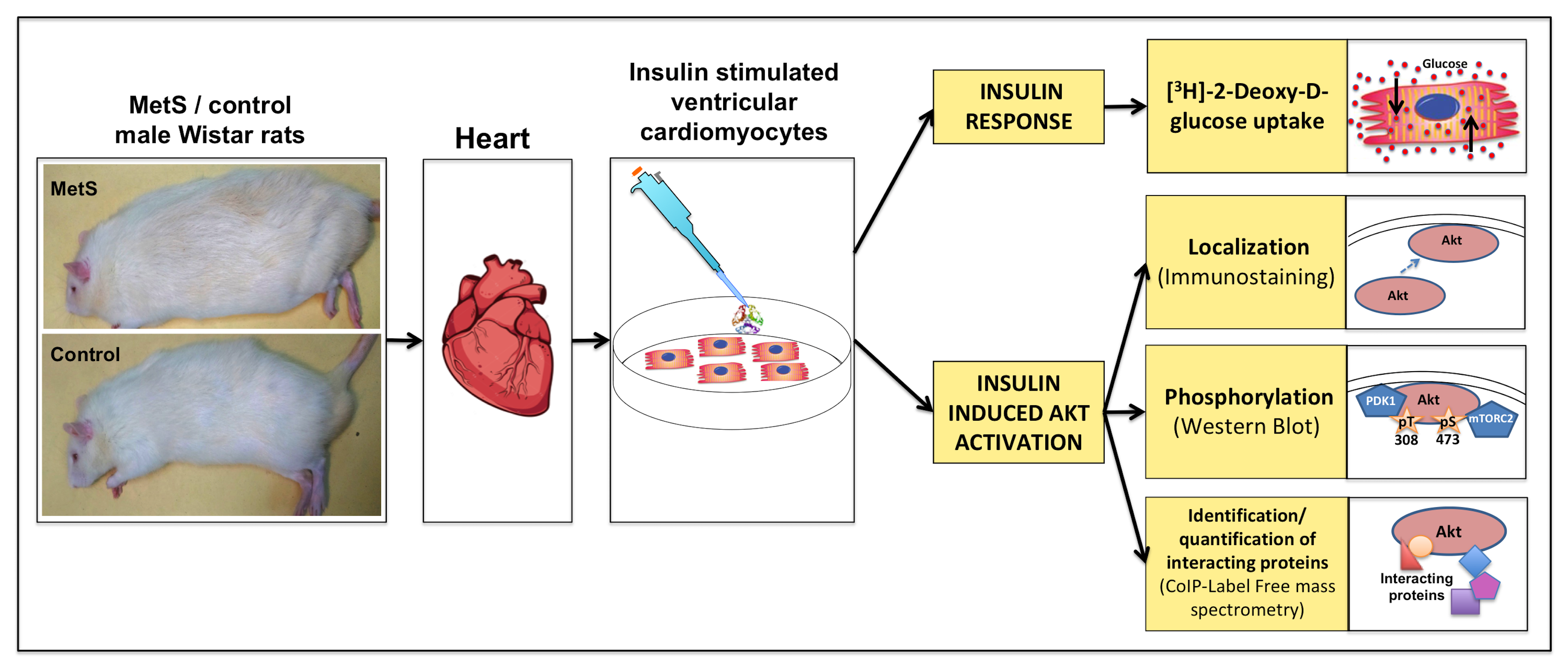

Supplement: S1 Fig — Male Wistar rats received tap water (control group) or 30% sucrose as drinking water (MetS group) for 4 months. Cardiomyocytes were isolated and insulin response was assessed by [3H]-2-Deoxy-D-glucose uptake assays. To evaluate insulin-induced Akt activation, the localization of this kinase was determined by immunostaining and its phosphorylation in the two main activation residues, serine 473 and threonine 308, by Western Blot. Identification and relative abundance of proteins interacting with Akt were evaluated by label-free mass spectrometry, in order to provide proteomic support to its activation status and the insulin resistance state of the cardiomyocytes. (TIF) [file pone.0228115.s001.tif]

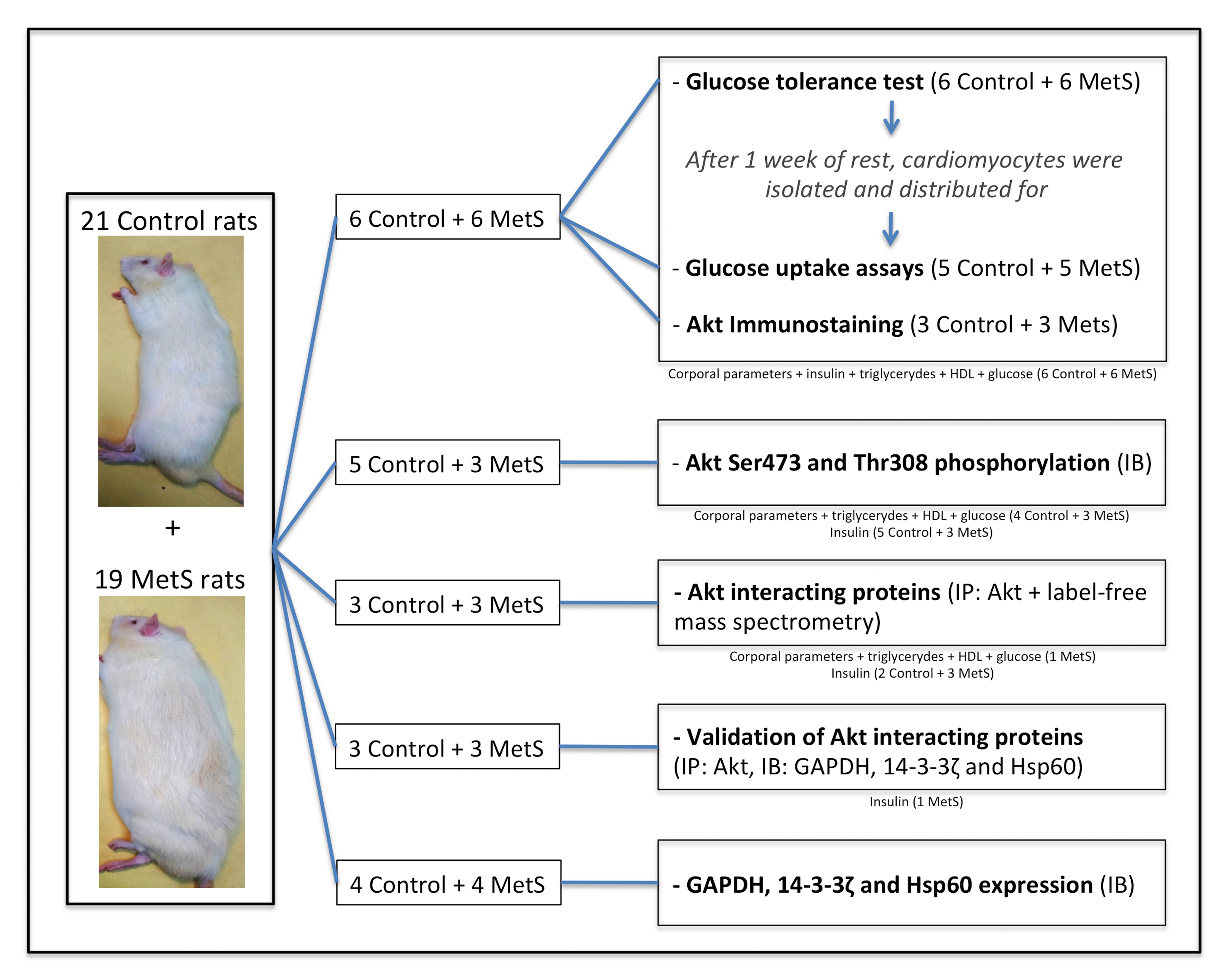

Supplement: S2 Fig — Scheme depicting the number of control and MetS rats distributed over the performed analyses. (TIF) [file pone.0228115.s002.tif]

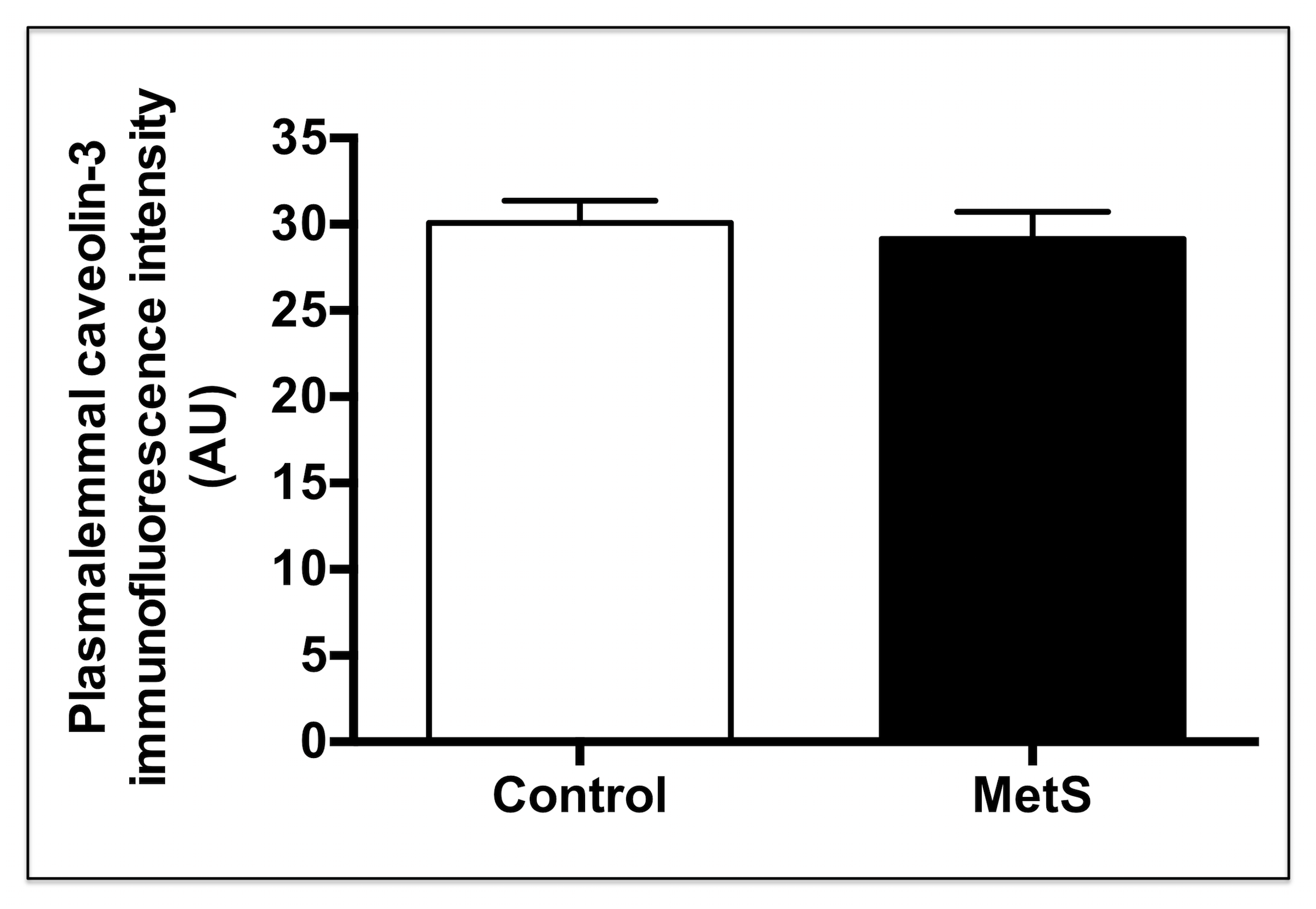

Supplement: S3 Fig — Bar chart representing average caveolin-3 immunofluorescence intensity in 10 regions of interest (ROIs) with an area of 10 μm2, selected in the plasmalemmal region of 10 cardiomyocytes (10 ROIs per cell) from each experimental condition. AU = arbitrary units. (TIF) [file pone.0228115.s003.tif]

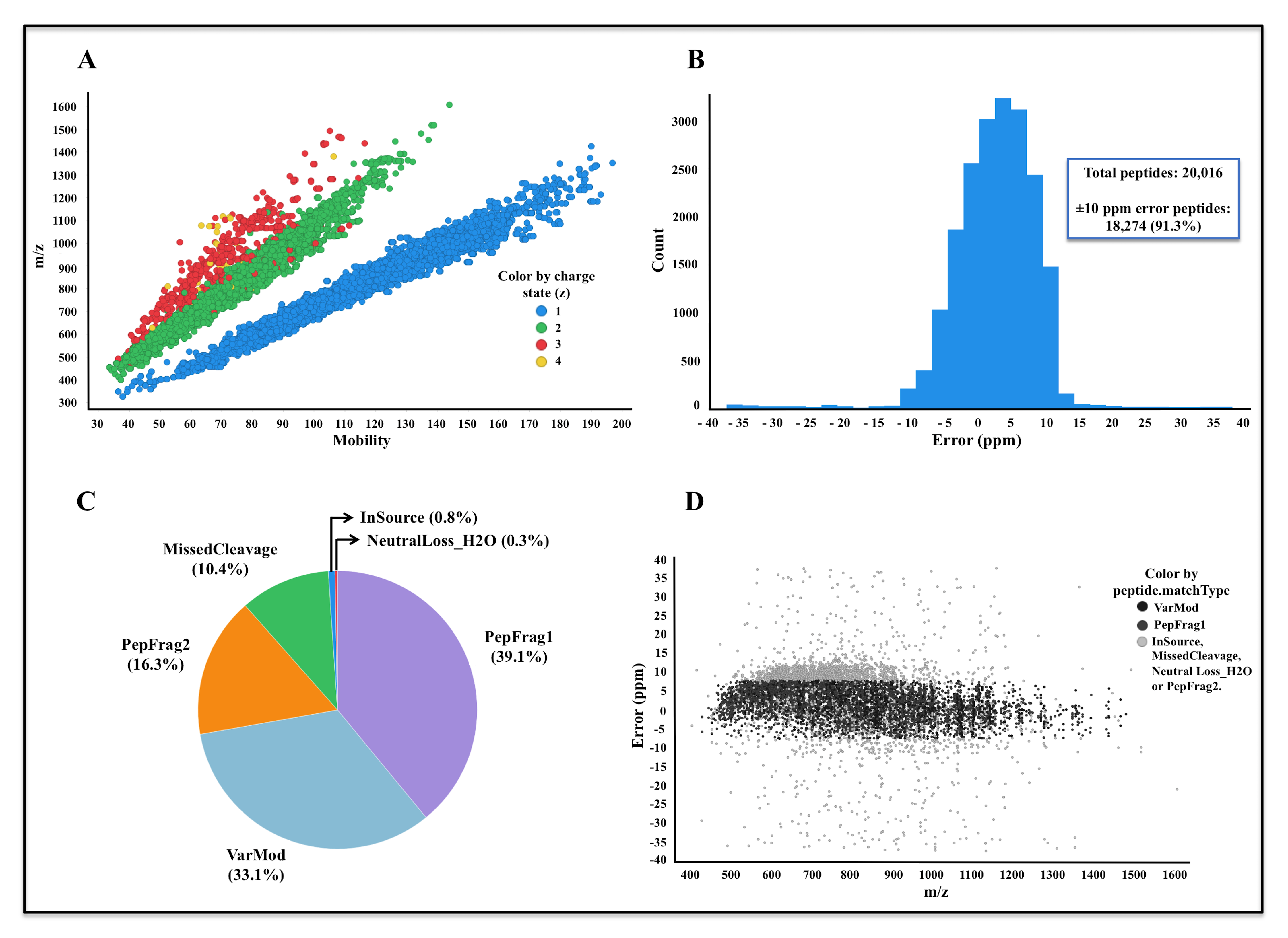

Supplement: S4 Fig — A) Movement of the ions inside of the mobility cell. Ions with charge state z = 1+ (blue dots) were discarded in this study, only ions with charge state of z = 2+ or higher (peptides indicated by green, red and yellow dots) were used to identified and quantify proteins. B) Histogram representing 20,016 peptides which 91.3% had an error of ±10 ppm, no peptide used for the identification and quantification of proteins exceeded ± 38 ppm. C) Peptide match type classification. PepFrag 1 peptides and VarMod peptides represent 72.2% of the total ions. D) PepFrag 1 and VarMod peptides (black dots) are distributed through the full m/z range, in no more than ± 10 ppm. These peptides are the most reliable for protein identification. (TIF) [file pone.0228115.s004.tif]

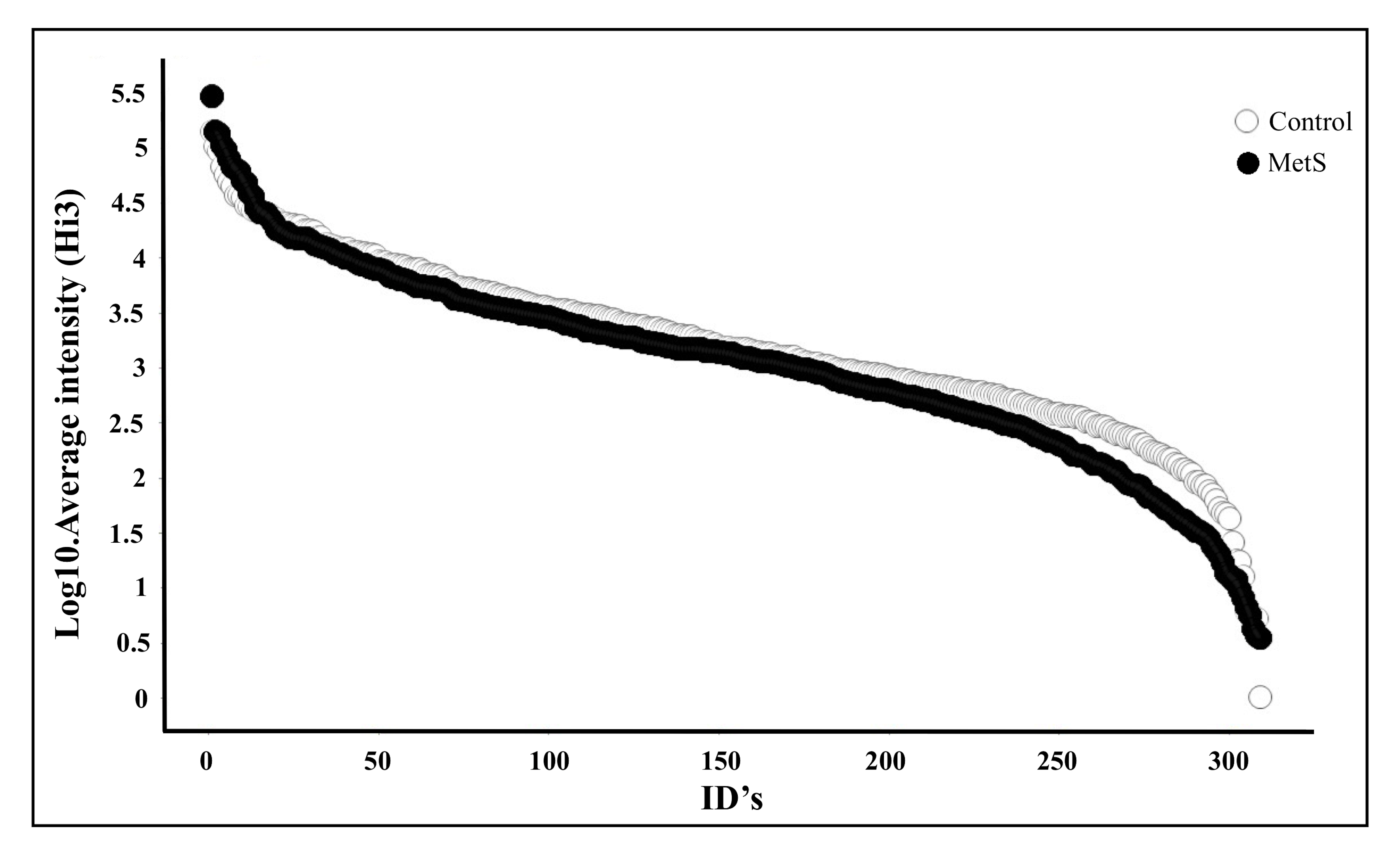

Supplement: S5 Fig — In white are represented the proteins in the control sample and in black the proteins in MetS. X-axis corresponds to the number of identified and quantified proteins (ID’s); Y-axis corresponds to the average of the Hi3 intensities in the technical triplicate for each detected protein (values are represented as Log10). (TIF) [file pone.0228115.s005.tif]

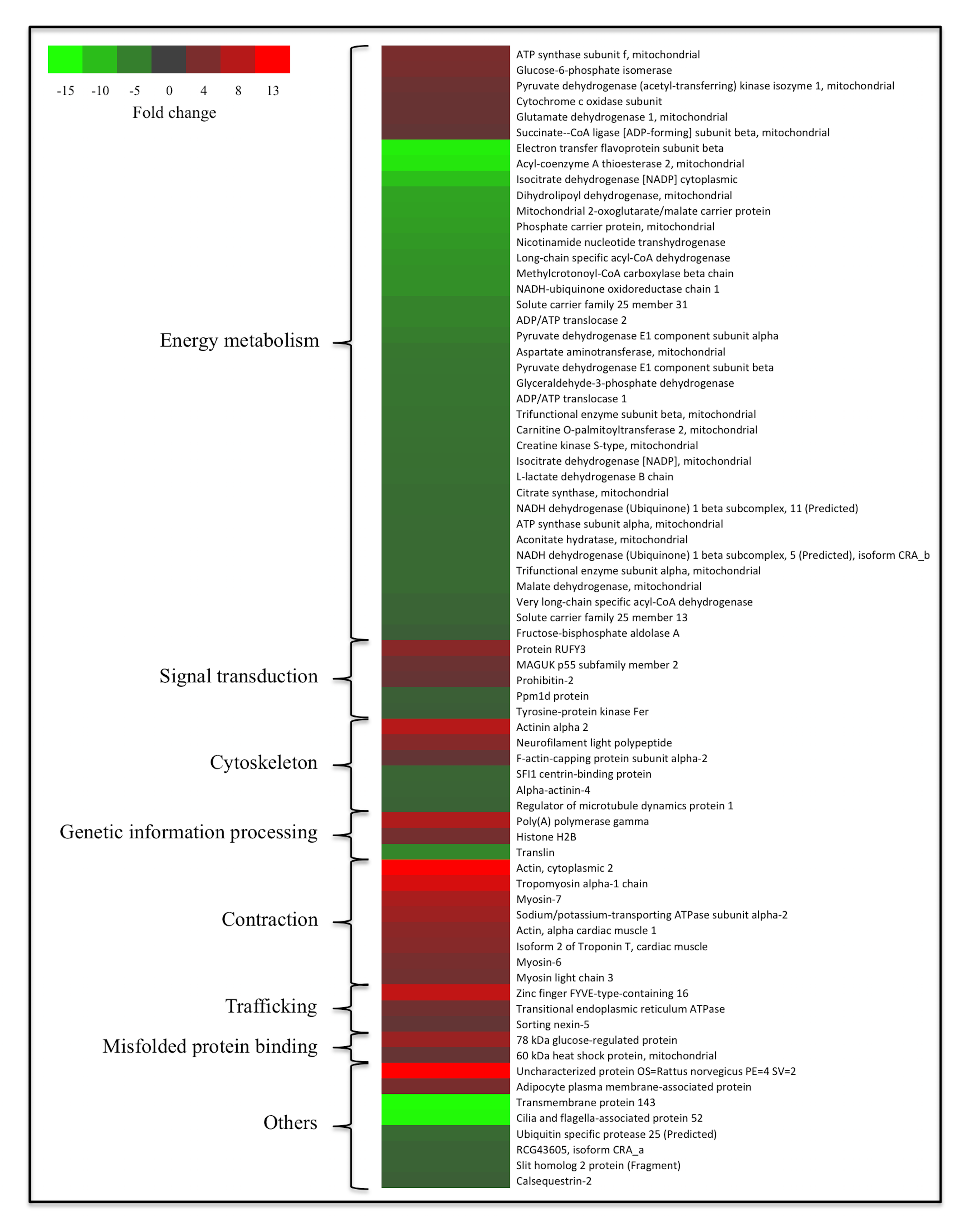

Supplement: S6 Fig — Heat map representation of proteins with abundance changes in Akt immunocomplexes in MetS cardiomyocytes vs control. Proteins are classified by main physiological function. (TIF) [file pone.0228115.s006.tif]
