# Supplementary material for: Metabolic syndrome diminishes insulin-induced Akt activation and causes a redistribution of Akt-interacting proteins in cardiomyocytes
Source: PLoS One. 2020 Jan 29;15(1):e0228115. doi: 10.1371/journal.pone.0228115 (PMC6988918; doi:10.1371/journal.pone.0228115)
Supplement: S1 Table — (DOCX) [file pone.0228115.s008.docx]

**S4 Table. Proteins with unchanged interaction with Akt in MetS cardiomyocytes.**

| **Accession** | **Description** | **Peptide count** | **Unique peptides** | **Confidence score** |
| --- | --- | --- | --- | --- |
| P34058 | Heat shock protein HSP 90-beta | 3 | 2 | 22.0 |
| A0A0G2K808 | ArfGAP with SH3 domain, ankyrin repeat and PH domain 2 | 3 | 3 | 13.0 |
| P81155 | Voltage-dependent anion-selective channel protein 2 | 19 | 12 | 97.1 |
| P32551 | Cytochrome b-c1 complex subunit 2, mitochondrial | 68 | 65 | 317.4 |
| Q68FY0 | Cytochrome b-c1 complex subunit 1, mitochondrial | 52 | 49 | 233.8 |
| D3ZLT1 | NADH dehydrogenase (Ubiquinone) 1 beta subcomplex, 7 (Predicted) | 5 | 5 | 32.1 |
| Q5XIH3 | NADH dehydrogenase [ubiquinone] flavoprotein 1, mitochondrial | 38 | 36 | 191.3 |
| P68370 | Tubulin alpha-1A chain | 4 | 4 | 23.2 |
| P12847 | Myosin-3 | 77 | 6 | 355.9 |
| Q5PQZ9 | NADH dehydrogenase [ubiquinone] 1 subunit C2 | 4 | 4 | 22.8 |
| Q5QJC9 | BAG family molecular chaperone regulator 5 | 2 | 1 | 10.0 |
| D3ZUX5 | MICOS complex subunit | 4 | 2 | 26.0 |
| Q66HF1 | NADH-ubiquinone oxidoreductase 75 kDa subunit, mitochondrial | 108 | 105 | 457.1 |
| P62804 | Histone H4 | 12 | 11 | 50.1 |
| P85834 | Elongation factor Tu, mitochondrial | 14 | 13 | 88.0 |
| Q29RW1 | Myosin-4 | 105 | 2 | 509.2 |
| D3ZG43 | NADH dehydrogenase (Ubiquinone) Fe-S protein 3 (Predicted), isoform CRA_c | 28 | 26 | 84.3 |
| Q64119 | Myosin light polypeptide 6 | 7 | 3 | 44.9 |
| B0BNE6 | NADH dehydrogenase (Ubiquinone) Fe-S protein 8 (Predicted), isoform CRA_a | 9 | 7 | 49.7 |
| F1LX07 | Solute carrier family 25 member 12 | 19 | 11 | 94.1 |
| P20788 | Cytochrome b-c1 complex subunit Rieske, mitochondrial | 8 | 7 | 63.0 |
| P35435 | ATP synthase subunit gamma, mitochondrial | 14 | 13 | 51.9 |
| B6RK61 | Myosin heavy chain 7B | 31 | 11 | 153.3 |
| Q6AXV4 | Sorting and assembly machinery component 50 homolog | 16 | 14 | 120.3 |
| P11530 | Dystrophin | 6 | 4 | 27.9 |
| P04462 | Myosin-8 | 66 | 2 | 280.1 |
| D3ZFQ8 | Cytochrome c-1 | 22 | 20 | 103.3 |
| Q561S0 | NADH dehydrogenase [ubiquinone] 1 alpha subcomplex subunit 10, mitochondrial | 32 | 30 | 161.6 |
| Q3KR86 | MICOS complex subunit MIC60 | 29 | 27 | 202.9 |
| P04764 | Alpha-enolase | 6 | 4 | 37.4 |
| B5DEL8 | NADH dehydrogenase (Ubiquinone) Fe-S protein 5 | 8 | 1 | 41.3 |
| D3ZZ21 | NADH dehydrogenase (Ubiquinone) 1 beta subcomplex, 6 (Predicted) | 3 | 3 | 27.3 |
| P00406 | Cytochrome c oxidase subunit 2 | 28 | 24 | 168.4 |
| P06685 | Sodium/potassium-transporting ATPase subunit alpha-1 | 10 | 4 | 55.2 |
| P23928 | Alpha-crystallin B chain | 4 | 4 | 28.5 |
| P02563 | Myosin-6 | 410 | 3 | 2175.7 |
| Q9Z2L0 | Voltage-dependent anion-selective channel protein 1 | 39 | 35 | 177.0 |
| B2RYS8 | NADH dehydrogenase [ubiquinone] 1 | 5 | 5 | 17.4 |
| P15651 | Short-chain specific acyl-CoA dehydrogenase, mitochondrial | 5 | 5 | 28.0 |
| P19234 | NADH dehydrogenase [ubiquinone] flavoprotein 2, mitochondrial | 17 | 15 | 96.7 |
| P28064 | Proteasome subunit beta type-8 | 3 | 1 | 9.5 |
| P63018 | Heat shock cognate 71 kDa protein | 6 | 3 | 34.0 |
| P52504 | NADH dehydrogenase [ubiquinone] iron-sulfur protein 6, mitochondrial | 4 | 3 | 13.0 |
| D3ZE15 | NADH dehydrogenase [ubiquinone] 1 alpha subcomplex subunit 13-like | 16 | 14 | 84.0 |
| Q6P0K8 | Junction plakoglobin | 4 | 2 | 17.7 |
| B2RYS2 | Cytochrome b-c1 complex subunit 7 | 6 | 6 | 48.4 |
| P10888 | Cytochrome c oxidase subunit 4 isoform 1, mitochondrial | 31 | 28 | 144.0 |
| F1LWG8 | Sarcalumenin | 11 | 10 | 56.7 |
| P14408-2 | Isoform Cytoplasmic of Fumarate hydratase, mitochondrial | 6 | 5 | 37.5 |
| Q9QZ76 | Myoglobin | 5 | 5 | 32.4 |
| D3ZKZ5 | Tuftelin 1 | 3 | 2 | 14.9 |
| Q9Z1P2 | Alpha-actinin-1 | 7 | 2 | 40.3 |
| D4A3V2 | NADH dehydrogenase [ubiquinone] 1 alpha subcomplex subunit 6 | 13 | 13 | 78.5 |
| P12075 | Cytochrome c oxidase subunit 5B, mitochondrial | 12 | 11 | 59.4 |
| D4A4P3 | NADH:ubiquinone oxidoreductase subunit B3 | 2 | 2 | 13.3 |
| A0A0G2JUG1 | Uncharacterized protein | 15 | 3 | 65.1 |
| P15429 | Beta-enolase | 3 | 2 | 18.0 |
| A0A0G2JZJ9 | Uncharacterized protein | 9 | 2 | 40.9 |
| Q07969 | Platelet glycoprotein 4 | 3 | 2 | 19.1 |
| Q7TQ16 | Cytochrome b-c1 complex subunit 8 | 5 | 5 | 21.9 |
| P11240 | Cytochrome c oxidase subunit 5A, mitochondrial | 11 | 9 | 64.8 |
| D4A0T0 | NADH:ubiquinone oxidoreductase subunit B10 | 16 | 16 | 62.2 |
| P10719 | ATP synthase subunit beta, mitochondrial | 62 | 54 | 330.9 |
| P11951 | Cytochrome c oxidase subunit 6C-2 | 5 | 5 | 23.0 |
| P14604 | Enoyl-CoA hydratase, mitochondrial | 5 | 4 | 31.1 |
| P08461 | Dihydrolipoyllysine-residue acetyltransferase component of pyruvate dehydrogenase complex, mitochondrial | 11 | 11 | 70.2 |
| Q5I0F1 | BRCA1-A complex subunit Abraxas 1 | 3 | 2 | 14.8 |
| P41350 | Caveolin-1 | 3 | 1 | 20.8 |
| Q63362 | NADH dehydrogenase [ubiquinone] 1 alpha subcomplex subunit 5 | 4 | 4 | 20.0 |
