# Supplementary material for: Metabolic syndrome diminishes insulin-induced Akt activation and causes a redistribution of Akt-interacting proteins in cardiomyocytes
Source: PLoS One. 2020 Jan 29;15(1):e0228115. doi: 10.1371/journal.pone.0228115 (PMC6988918; doi:10.1371/journal.pone.0228115)
Supplement: S1 Raw Images — (PDF) [file pone.0228115.s009.pdf]

Fig 4A

Control rat cardiomyocytes

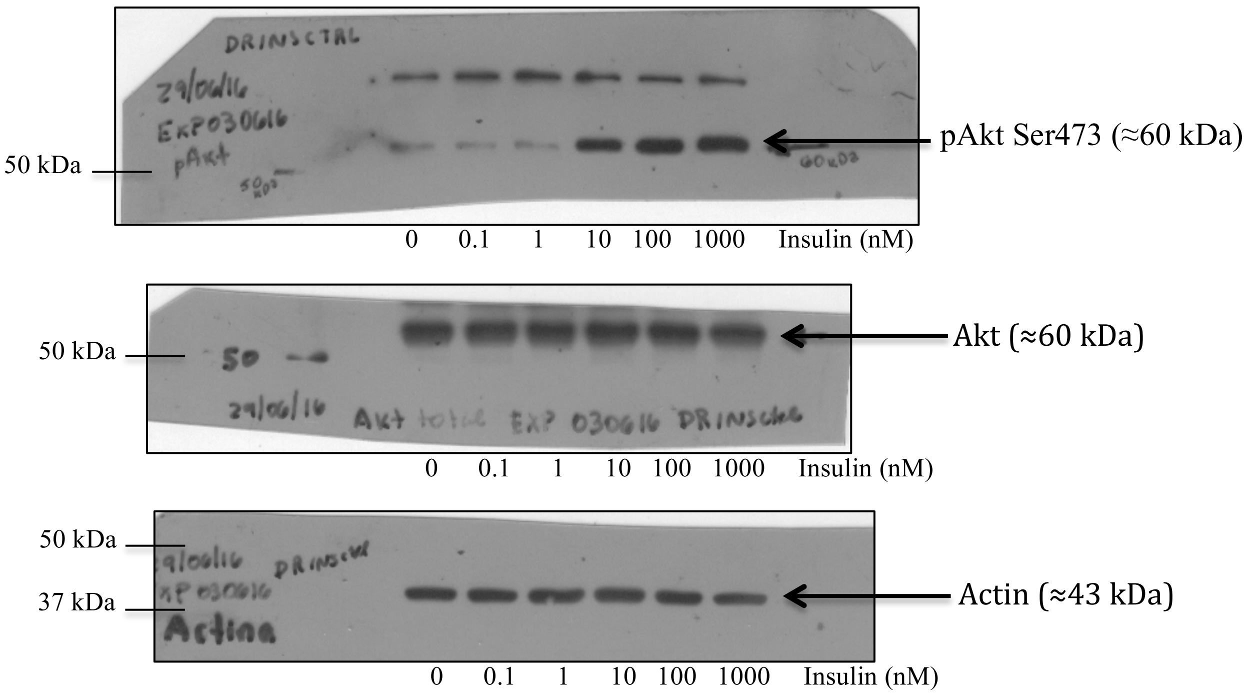

Metabolic syndrome rat cardiomyocytes

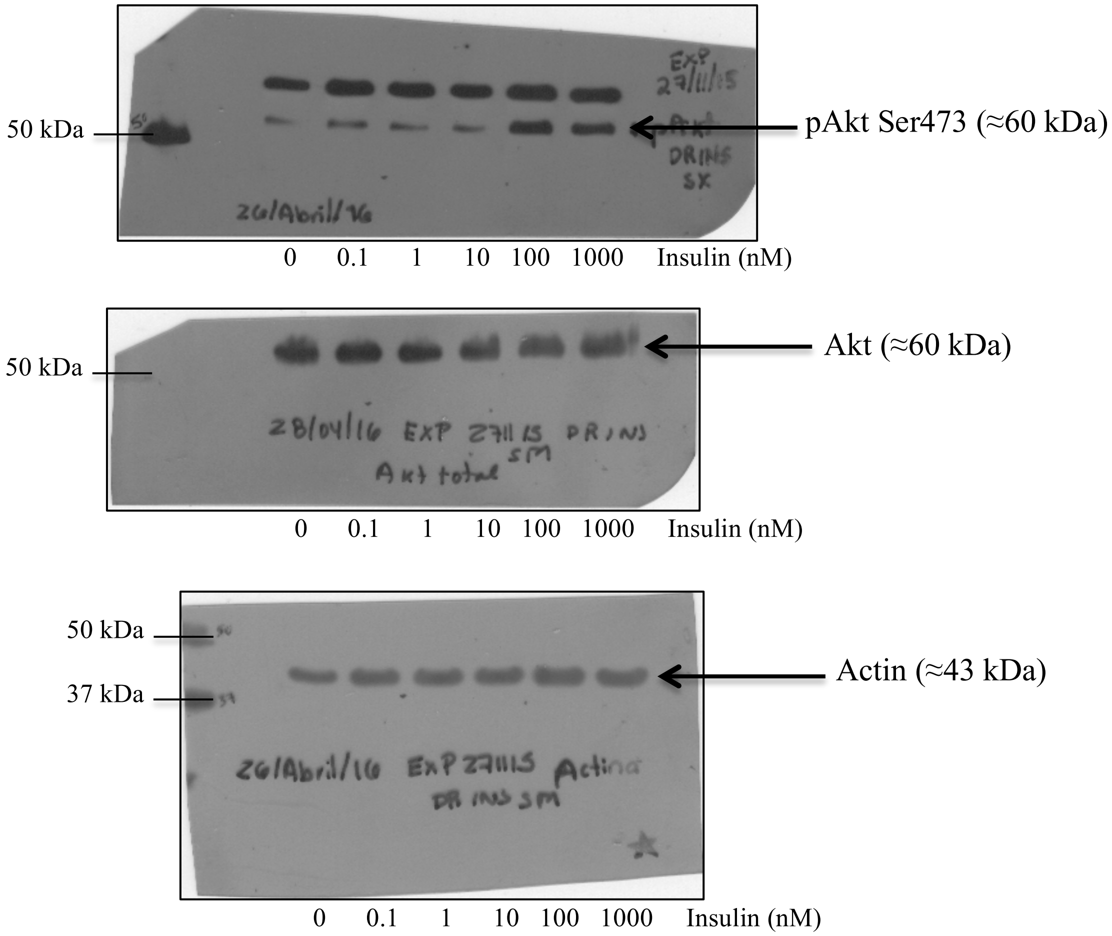

Fig 4B

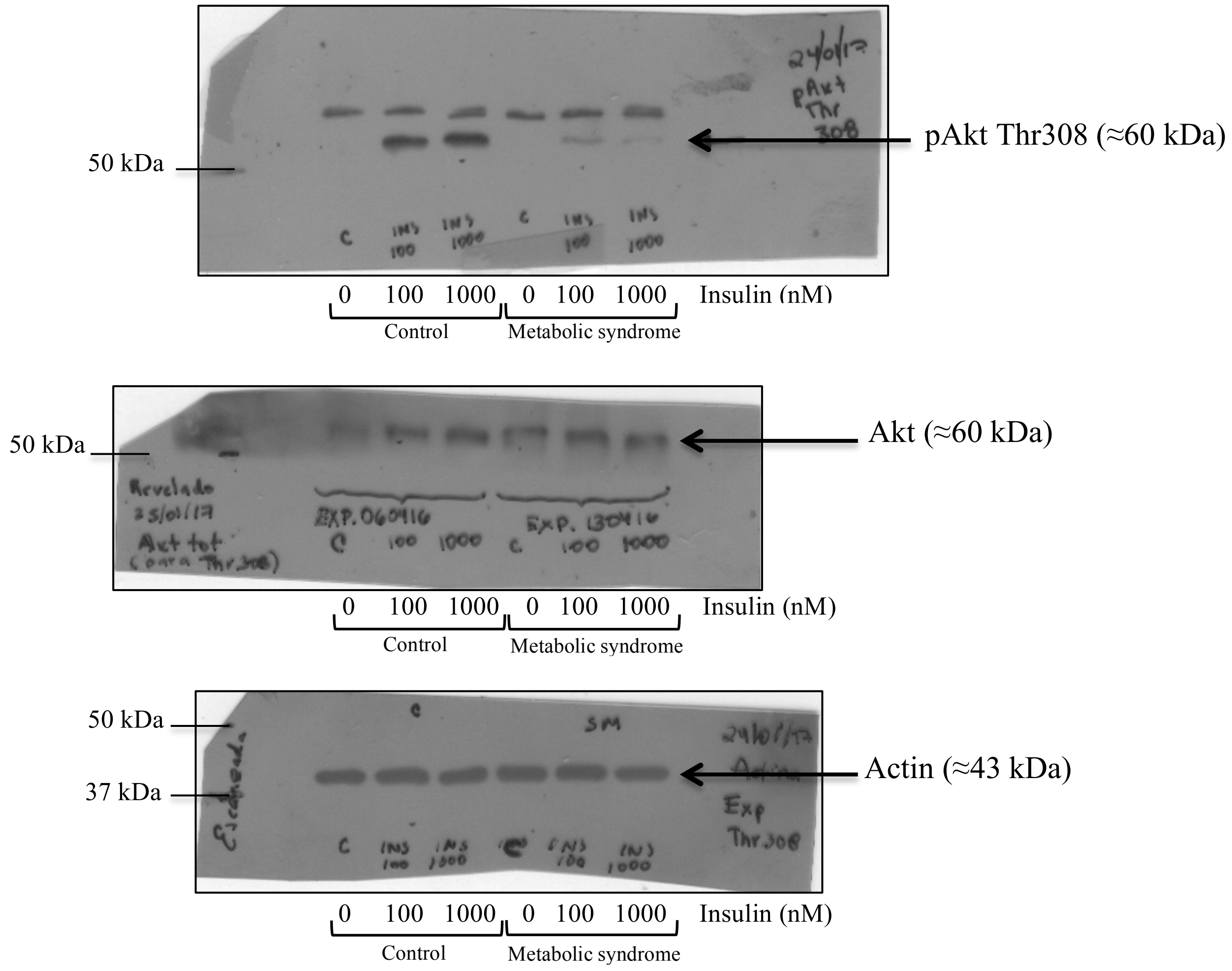

50 kDa
pAkt Thr308 (≈60 kDa)
0 100 1000 0 100 1000 Insulin (nM)
Control
Metabolic syndrome
50 kDa
Akt (≈60 kDa)
0 100 1000 0 100 1000 Insulin (nM)
Control
Metabolic syndrome
50 kDa
37 kDa
Actin (≈43 kDa)
0 100 1000 0 100 1000 Insulin (nM)
Control
Metabolic syndrome

Fig 6A

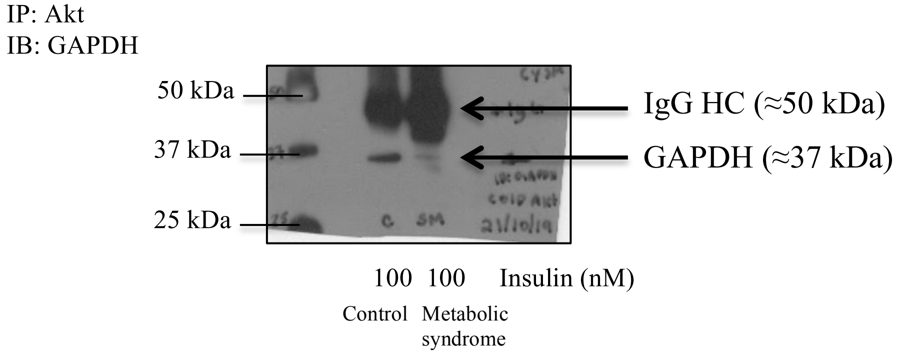

IP: Akt
IB: GAPDH
50 kDa
37 kDa
25 kDa
IgG HC (≈50 kDa)
GAPDH (≈37 kDa)
100 100 Insulin (nM)
Control
Metabolic syndrome

Fig 6B

GAPDH expression

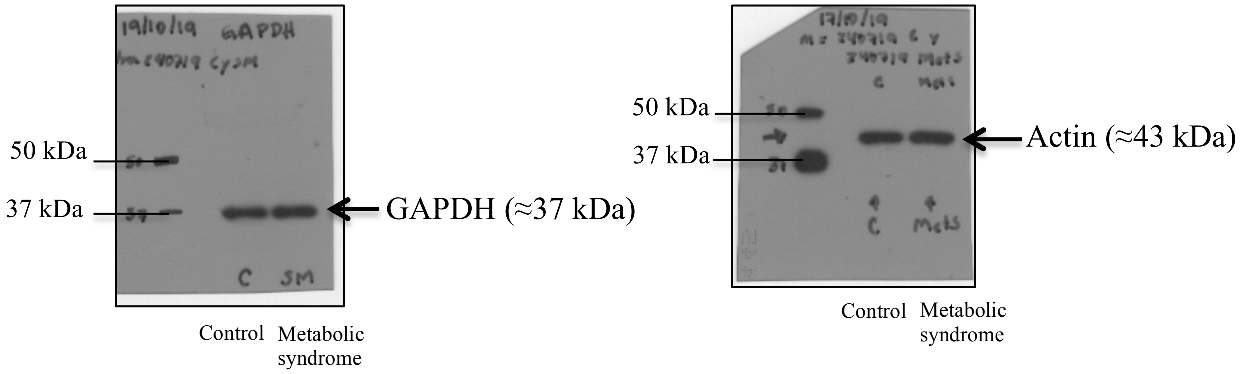

Same samples were used in representative blots shown in Figures 6B, 6D and 6F. Actin blot (loading control) is the same for the three panels, as indicated in figure legend.

Fig 6C

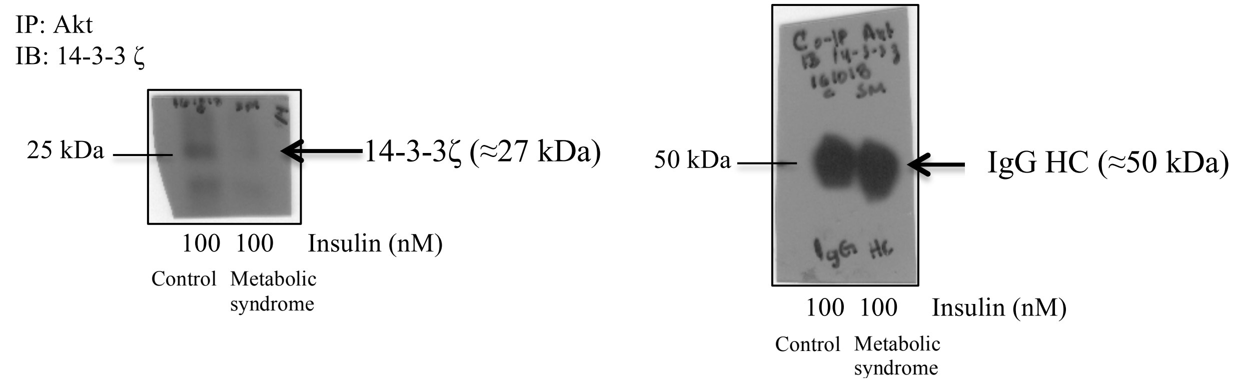

Fig 6D

14-3-3ζ expression

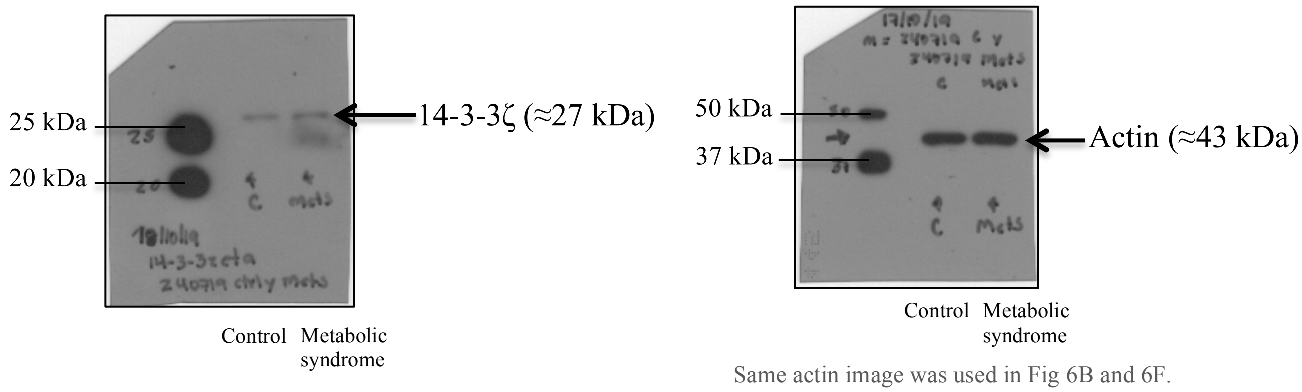

Same actin image was used in Fig 6B and 6F.

Fig 6E

IP: Akt
IB: Hsp60

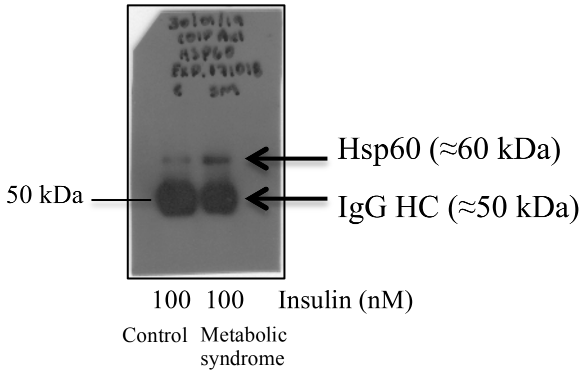

Fig 6F

Hsp60 expression

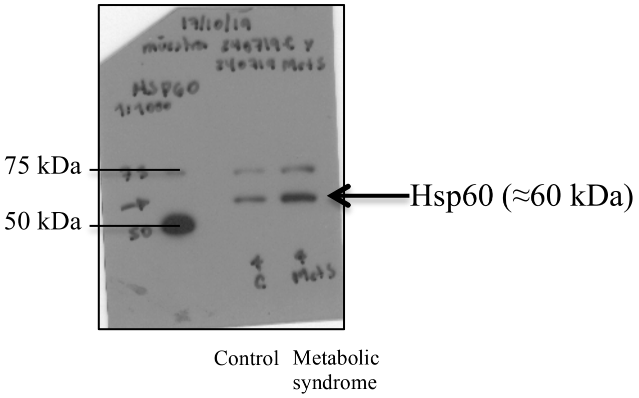

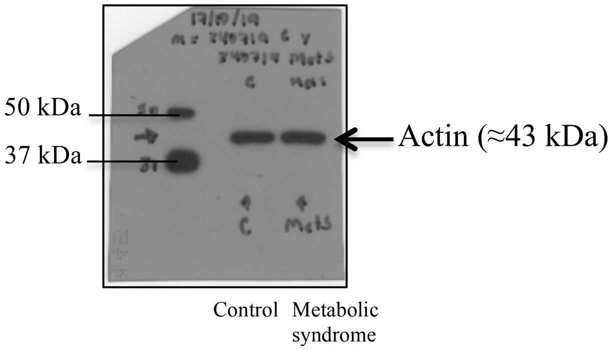

Same actin image was used in Fig 6B and 6D.
